# Effects of bystander interventions on school bullying: a systematic review and meta-analysis

**DOI:** 10.1590/1980-220X-REEUSP-2026-0029en

**Published:** 2026-11-02

**Authors:** Luiza Luana de Araújo Lira Bezerra, Raquel Sampaio Florêncio, Dayse Lorrane Gonçalves Alves, Valter Cordeiro Barbosa, Thereza Maria Magalhães Moreira, Edna Maria Camelo Chaves, Mirna Albuquerque Frota, Bruna Rodrigues Nunes, Thiago Santos Garces, Açucena Leal de Araújo, Ilvana Lima Verde Gomes

**Affiliations:** 1 Universidade Estadual do Ceará, Centro de Ciências da Saúde, Programa de Pós-Graduação em Saúde Coletiva, Fortaleza, CE, Brazil.; 2 Universidade Estadual do Ceará, Centro de Ciências da Saúde, Programa de Pós-Graduação Cuidados Clínicos em Enfermagem e Saúde, Fortaleza, CE, Brazil.; 3 Universidade de Fortaleza, Programa de Pós-graduação em Saúde Coletiva, Fortaleza, CE, Brazil.; 4 Universidade Regional do Cariri, Centro de Ciências da Saúde, Departamento de Enfermagem, Crato, CE, Brazil.

**Keywords:** Bullying, Cyberbullying, Schools, Systematic review, Bullying, Cyberbullying, Instituições Acadêmicas, Revisão Sistemática

## Abstract

**Objective:**

To synthesize evidence on the effectiveness of school-based bystander interventions in reducing bullying and cyberbullying among children and adolescents.

**Method:**

This systematic review and meta-analysis followed PRISMA 2020 and was registered in PROSPERO (CRD42021244706). Experimental and quasi-experimental controlled studies were identified through databases, gray literature, reference lists, and reviews. Independent reviewers selected studies, extracted data, and assessed quality using the Downs and Black checklist. Random-effects meta-analyses estimated risk ratios (RRs) and mean differences; certainty was assessed with GRADE.

**Results:**

Twenty-three studies were included, with samples ranging from 54 to 23,430 participants. Interventions reduced traditional bullying victimization (RR 0.63, 95% CI 0.40–0.99; p = 0.04; I2 = 0%) and perpetration (RR 0.75, 95% CI 0.60–0.95; p = 0.02; I2 = 0%). Effects were not significant for cyberbullying perpetration or defensive bystander behavior. Certainty was high for traditional bullying outcomes and very low for the remaining outcomes.

**Conclusion:**

Interventions with a bystander component may reduce traditional bullying perpetration and victimization, but evidence for cyberbullying and defensive behavior remains limited.

## RESUMO

**Objetivo:**

Sintetizar evidências sobre a eficácia de intervenções do espectador no ambiente escolar para reduzir o bullying e o cyberbullying entre crianças e adolescentes.

**Método:**

Esta revisão sistemática e metanálise seguiu as diretrizes PRISMA 2020 e foi registrada no PROSPERO (CRD42021244706). Estudos controlados experimentais e quase-experimentais foram identificados por meio de bases de dados, literatura cinzenta, listas de referências e revisões. Revisores independentes selecionaram os estudos, extraíram os dados e avaliaram a qualidade usando a lista de verificação de Downs e Black. Metanálises de efeitos aleatórios estimaram as razões de risco (RR) e as diferenças médias; a certeza foi avaliada com o sistema GRADE.

**Resultados:**

Foram incluídos 23 estudos, com amostras que variaram de 54 a 23.430 participantes. As intervenções reduziram a vitimização por bullying tradicional (RR 0,63, IC 95% 0,40–0,99; p = 0,04; I2 = 0%) e a perpetração (RR 0,75, IC 95% 0,60–0,95; p = 0,02; I2 = 0%). Os efeitos não foram significativos para a perpetração de cyberbullying ou para o comportamento defensivo de espectadores. O grau de certeza foi alto para os desfechos relacionados ao bullying tradicional e muito baixo para os demais desfechos.

**Conclusão:**

Intervenções que incluam a participação de espectadores podem reduzir a perpetração e a vitimização do bullying tradicional, mas as evidências para o cyberbullying e o comportamento defensivo ainda são limitadas.

## INTRODUCTION

Bullying, a prevalent public health concern, profoundly impacts mental and physical health, increasing the risk of anxiety, depression, and suicidal ideation, with effects that may persist into adulthood. Consequently, bullying prevention is a priority in health and education policies.

While school-based antibullying interventions show small to moderate effects on reducing perpetration and victimization, long-term efficacy remains unclear^(1–4)^. Traditionally, these interventions focus on victims and aggressors and may underemphasize the role of bystanders^(5)^. However, bullying is a group phenomenon, and bystanders can influence its dynamics^(6)^. Passive observation may reinforce bullying, whereas active intervention may help interrupt it.

Despite evidence emphasizing the importance of bystander interventions^(7–10)^, research directly evaluating their impact on bullying prevalence remains limited^(11–13)^. A meta-analysis of 12 studies (12,784 participants) found modest positive effects (Hedges g = 0.20, 95% CI: 0.11–0.29, p-value < 0.001) of antibullying interventions on increasing bystanders’ defensive behavior, with stronger effects in high school students (ES = 0.43) than in younger students (ES = 0.14, p-value < 0.05). However, this review, limited to studies published between 1998 and 2010, focused on defensive behavior and empathy, rather than directly assessing reductions in bullying or cyberbullying outcomes. Given the recognized role of bystanders in prevention, this systematic review aims to synthesize current evidence on the effectiveness of school-based interventions targeting bystanders to reduce bullying.

## METHOD

### Protocol and Registration

This systematic review was reported according to the Preferred Reporting Items for Systematic Reviews and Meta-Analyses (PRISMA 2020)^(14)^ and conducted following Cochrane methodological guidance^(15)^. The protocol was registered in the International Prospective Register of Systematic Reviews (PROSPERO; CRD42021244706).

### Research Question and Eligibility Criteria

The PICO framework^(16,17)^ guided the question: “What is the effect of school-based interventions targeting bystanders on bullying?” Eligible reports evaluated school-based programs focused on bystander involvement in bullying or cyberbullying among children and adolescents of any gender; used an experimental or quasi-experimental controlled design; and reported at least one prespecified outcome: bystander intervention or defending behavior, bullying victimization, bullying perpetration, cyberbullying perpetration, or cybervictimization. Peer-reviewed articles, book chapters, theses, and dissertations were eligible without date or language restrictions. For synthesis, studies were grouped by outcome construct, data type (dichotomous or continuous), intervention exposure, assessment time point, and the availability of clinically comparable group data.

### Sources of Information

The search was last updated in June 2025 in PubMed, Web of Science, Scopus, SciELO, Embase, LILACS, CINAHL, ERIC, the Cochrane Central Register of Controlled Trials, ScienceDirect, and PsycArticles. Gray-literature sources included the Brazilian Digital Library of Theses and Dissertations, the CAPES Theses and Dissertations Catalog, ProQuest Dissertations and Theses, and Google Scholar. Reference lists of included studies and relevant systematic reviews and meta-analyses were also searched manually.

### Search Strategies

The primary strategy (S1) combined controlled vocabularies (DeCS, MeSH, and Emtree) and free-text terms organized according to PICOS, without a comparator search block. Because the full strategy was not compatible with ERIC, ScienceDirect, LILACS, SciELO, Google Scholar, ProQuest Dissertations and Theses, the CAPES catalog, and PsycArticles, simplified keyword combinations (A1–A4) developed with librarian support were used in those sources. Charts 1 and 2 present the complete strategies.

**Chart 1:** Search strategy (S1) by groups and descriptors (PICOS) – Fortaleza, CE, Brazil, 2025.

Group of descriptors	Descriptors
Population	(child OR children OR childhood OR boy OR girl OR “school child” OR “school boy” OR “school children” OR “school girl” OR “school-going” OR schoolboy OR schoolchild OR schoolchildren OR schoolgirl OR schoolgirls OR schoolgoing OR adolescent OR adolescence OR teen OR teenager OR youth OR student OR “elementary student” OR “elementary pupils” OR “elementary school pupil” OR “elementary school student“ OR “high school student” OR “middle school student” OR school OR “educational institution” OR “primary school” OR “secondary school” OR “high school” OR “middle school” OR “elementary school” OR “school-aged”)
Intervention	(witness OR “witness corroboration” OR “eye witness” OR eyewitness OR testimony OR “bullying witnessed” OR witnesses OR witnessed OR “witnessing bullying” OR “bullying witnessed” OR “bullying witnessing” OR bystander OR spectator)
Comparison	Control/usual practice/no intervention; assessed during eligibility and not used as a search term
Outcome	(bullying OR cyberbullying OR “cyber-bullying” OR “e-bullying” OR “electronic bullying” OR “internet bullying” OR “online bullying” OR aggression OR “aggressive attitude” OR “aggressive behavior” OR “aggressive behaviour” OR “aggressive reaction” OR aggressiveness OR aggressivity OR “counter aggression” OR counteraggression OR provocation OR hostility OR “hostile verb” OR “psychological antagonism” OR threat OR “school bullying” OR “school victimization” OR “victimization among school” OR “bully victimization” OR “school bullying victimization” OR “bully-victim” OR “peer victimization” OR “peer abuse” OR “psychological victimization” OR “stigma experienced” OR “involvement in bullying” OR “peer harassment” OR “relational aggression” OR “peer rejection” OR “online harassment” OR “cyber-harassment” OR “cyberharassment” OR “electronic harassment” OR “internet harassment”)
Study design	(“Intervention Study” OR “intervention studies” OR “intervention trial” OR “interventional studies” OR “interventional study” OR “interventional trial” OR “quasi-randomised controlled trials” OR “controlled before-and-after” OR “Controlled Before After Studies” OR “Controlled Before-After Study” OR “CBA Studies” OR “Controlled Before and After Studies” OR “randomized controlled trial” OR “Randomized Controlled Trials as Topic” OR “randomised controlled study” OR “randomized controlled study” OR “controlled clinical trial” OR “controlled clinical comparison” OR “controlled clinical drug trial” OR “controlled clinical experiment” OR “controlled clinical study” OR “controlled clinical test” OR “Controlled Clinical Trials as Topic” OR “randomized controlled trials” OR “randomized controlled trial” OR “pragmatic clinical trials as topic” OR “randomized controlled trials” OR “randomized controlled trials as topic” OR “randomized controlled trial as topic” OR “Random Allocation” OR “Randomization” OR “random allocation” OR “randomisation” OR “double blind procedure” OR “double-blind method” OR “double blind clinical trial” OR “double blind comparison” OR “double blind design” OR “double blind studies” OR “double blind study” OR “double blind test” OR “double blind trial” OR “double masked clinical study” OR “double masked clinical trial” OR “double masked comparison” OR “double masked design” OR “double masked method” OR “double masked procedure” OR “double masked study” OR “double masked test” OR “double masked trial” OR “double-blind clinical study” OR “double blind method” OR “Double-Blind Methods” OR “Single-Blind Method” OR “single blind procedure” OR “single blind clinical study” OR “single blind clinical trial” OR “single blind comparison” OR “single blind design” OR “single blind studies” OR “single blind study” OR “single blind test” OR “single blind trial” OR “single masked clinical study” OR “single masked clinical trial” OR “single masked comparison” OR “single masked design” OR “single masked method” OR “single masked procedure” OR “single masked study” OR “single masked studies” OR “single masked test” OR “single masked trial” OR “single-blind method” OR “comparative study” OR “comparative studies” OR comparison OR “Evaluation Studies as Topic” OR evaluation OR “evaluation studies” OR “evaluation study” OR “Evaluation Report” OR “Evaluation Reports” OR Critique OR “Critiques” OR “Evaluation Research” OR “Use-Effectiveness” OR “Use Effectiveness” OR “Pre-Post Tests” OR “Pre Post Tests” OR “Qualitative Evaluation” OR “Qualitative Evaluations” OR “Quantitative Evaluation” OR “Quantitative Evaluations” OR “Theoretical Effectiveness” OR “Evaluation Indexes” OR “Evaluation Methodology” OR “Evaluation Methodologies” OR “Prospective Studies” OR “Prospective Study” OR “prospective method”)

**Chart 2:** A1, A2, A3, and A4 search strategies by keywords – Fortaleza, CE, Brazil, 2025.

Databases		Strategy by keywords
ScienceDirect	A1	school AND (child OR adolescent) AND bullying AND (witness OR bystander OR testimony) AND (“Intervention Study” OR "randomized controlled trial")
LILACS/Scielo/CTD-CAPES	A2	(school OR escola OR escuela) AND (child OR children OR criança OR niño OR adolescent OR adolescente) AND (bullying OR acoso)
ERIC/ProQuest/PsycArticles	A3	school AND (child OR adolescent) AND bullying AND (witness OR bystander OR testimony)
Google Scholar	A4	school AND (child OR adolescent) AND bullying AND (witness OR bystander OR testimony) AND (“randomized clinical trial” OR “clinical study”)

### Selection of Studies and Data Extraction

Two reviewers independently screened titles and abstracts and then assessed the full texts of potentially eligible reports. Disagreements were resolved by a third reviewer. Duplicate records were removed electronically and verified manually; no automation tool made eligibility decisions. Microsoft Excel worksheets were used to manage records and extract data. Three reviewers collected bibliographic information (authors, year, and country), study design, sample size and participant characteristics, intervention and comparator characteristics, delivery personnel, number and duration of sessions, follow-up, outcome definitions and measurement instruments, group-level summary statistics, effect estimates, and information needed for risk-of-bias assessment. Unclear information was checked against the full report and related publications. No outcome values were imputed; data were pooled only when the reported statistics were directly usable or could be derived from reported group data. Otherwise, findings were retained in the narrative synthesis.

### Risk of Bias Assessment

Methodological quality was assessed with the 27-item Downs and Black checklist across five domains^(18)^. This instrument was selected because the review included both randomized and nonrandomized controlled intervention studies, whereas RoB 2 is designed specifically for randomized trials and would not provide a single common framework for all eligible designs. Item 27 (power) was dichotomized as 1 for studies reporting at least 80% power and 0 otherwise. Total scores ranged from 0 to 28 and were classified as high risk (0–5)n, uncertain risk (6–18), or low risk (19–28)^(19)^. Two reviewers assessed each study independently, and a third reviewer resolved disagreements. Domain scores and the classification of every included study are presented in Chart 3.

**Chart 3:** Downs and Black domain scores and risk-of-bias classification for each included study – Fortaleza, CE, Brazil, 2025.

Study	Reporting (0–11)	External validity (0–3)	Internal bias (0–7)	Confounding (0–6)	Power (0–1)	Total (0–28)	Classification
Axford et al.^(38)^	11	1	6	5	1	24	Low
Chaux et al.^(20)^	9	1	4	5	0	19	Low
Desmet et al.^(23)^	11	2	5	5	1	24	Low
Kolic-Vehovec et al.^(35)^	9	1	5	4	0	19	Low
Karna et al.^(39)^	8	3	4	2	0	17	Uncertain
Menesini et al.^(37)^	5	2	3	1	1	12	Uncertain
Midgett et al.^(24)^	6	2	2	2	1	13	Uncertain
Midgett et al.^(25)^	9	2	4	3	0	18	Uncertain
Andreou et al.^(27)^	3	0	1	0	1	5	High
Karna et al.^(32)^	4	0	1	0	0	5	High
Stevens et al.^(29)^	9	3	5	5	0	22	Low
Williford et al.^(40)^	9	3	5	5	0	22	Low
Salmivalli et al.^(6)^	4	0	0	0	0	4	High
Midgett et al.^(26)^	6	1	4	3	0	14	Uncertain
Pack et al.^(36)^	1	0	0	0	0	1	High
Pfetsch et al.^(30)^	7	0	1	0	1	9	Uncertain
Saarento et al.^(34)^	8	3	4	4	0	19	Low
van der Ploeg et al.^(42)^	4	0	1	0	1	6	Uncertain
Yang and Salmivalli^(41)^	9	3	4	4	0	20	Low
Andreou et al.^(28)^	4	0	1	0	0	5	High
Nocentini and Menesini^(33)^	9	0	2	2	1	14	Uncertain
Vannini et al.^(31)^	4	0	1	0	0	5	High
Merrell^(22)^	6	1	4	3	0	14	Uncertain

Classification: 0–5, high risk; 6–18, uncertain risk; 19–28, low risk. Midgett et al.^(25)^ refers to the peer-advocate study; Midgett et al.^(26)^ refers to the junior-high-school study.

### Data Synthesis

A descriptive synthesis was prepared for all included studies. Random-effects meta-analysis was undertaken when studies were sufficiently comparable in population, intervention, outcome construct, measurement scale, and assessment time point. The first postintervention assessment was prioritized because follow-up schedules were sparse and heterogeneous. A study was counted once in descriptions of the evidence base, whereas independent nonoverlapping grade- or school-level strata could contribute separate comparisons to a meta-analysis. When Chaux et al.^(20)^ reported intervention arms of different durations, shorter- and longer-duration arms were evaluated in separate syntheses so that participants were not counted twice within the same pooled estimate.

Risk ratios (RRs) with 95% confidence intervals (CIs) were used for dichotomous outcomes, and mean differences (MDs) with 95% CIs were used for continuous outcomes. Dichotomous data were pooled with the Mantel-Haenszel method and continuous data with inverse-variance methods in Review Manager, using random-effects models.

Statistical heterogeneity was examined with Cochran’s Q, the chi-squared test, and I2. I2 values below 50% were interpreted as low, 50%–75% as moderate, and above 75% as high heterogeneity. Potential clinical sources were examined by comparing populations, intervention formats and durations, outcome definitions, measurement instruments, educational levels, and assessment timing across studies. Formal subgroup, meta-regression, and sensitivity analyses were not conducted as each synthesis contained few studies and outcome measurements were highly variable.

Risk of bias due to missing results was considered qualitatively by comparing reported methods and outcomes and by examining the completeness of results across reports. Since every meta-analysis contained fewer than 10 unique studies, funnel plots and statistical tests for small-study effects were not interpreted, as they would have had very low power.

### Evidence Certainty Ranking

The GRADE approach was used to rate certainty for each synthesis according to risk of bias, inconsistency, indirectness, imprecision, and other relevant considerations^(21)^. Certainty was classified as high, moderate, low, or very low. Two reviewers performed the assessment independently and resolved differences by consensus. The completed judgments are presented in the Results section.

## RESULTS

Electronic searches identified 3,134 records (2,866 from bibliographic databases and 268 from other electronic sources). After removal of 516 duplicates, 2,618 records were screened and 2,535 were excluded, leaving 83 reports for full-text assessment. Sixty-eight full-text reports were excluded and 15 met the eligibility criteria. Searches of previous systematic reviews and reference lists identified 780 additional records: 703 were excluded after title and abstract screening, 16 reports could not be retrieved, and 61 were assessed in full. Fifty-three were excluded, including 13 reports already identified through electronic searching, yielding eight additional eligible reports. Overall, 23 studies represented by 23 reports (22 journal articles and one thesis) were included (Figure 1).

**Figure 1:**
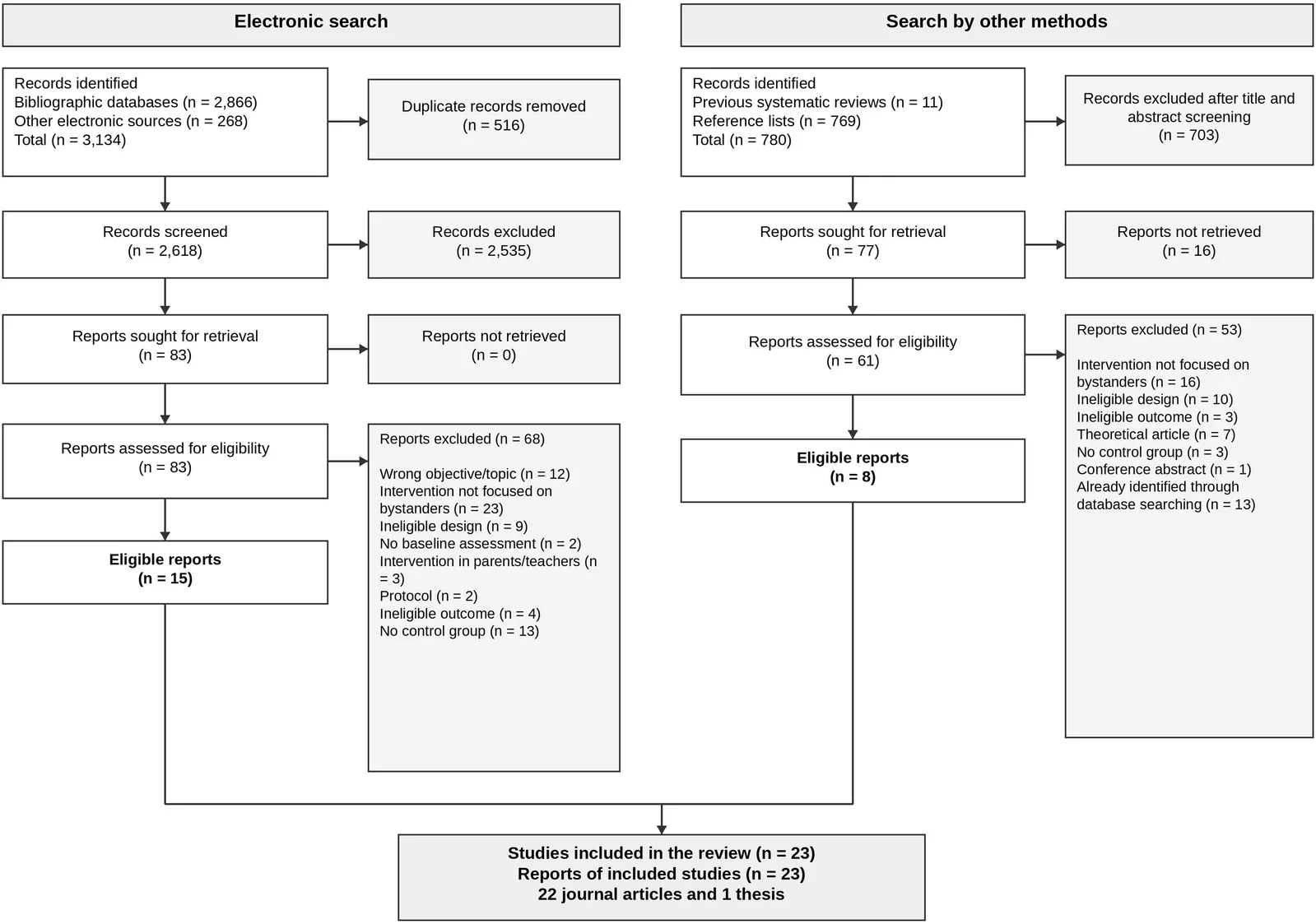
PRISMA 2020 flow diagram of study selection – Fortaleza, CE, Brazil, 2025.

### Aspects of Included Studies

The 23 included studies were published from 2000 to 2020. Finland contributed six studies (26.1%) and the United States five (21.7%). All reports were published in English, and 22 (95.7%) were journal articles. Seventeen studies (73.9%) used randomized controlled designs, and individual study samples ranged from 54 to 23,430 participants.

Intervention duration ranged from one to 20 months; two studies did not report duration^(20,22)^. Fourteen studies (60.9%) did not report follow-up. Among the nine studies with follow-up, the assessment occurred at one month in four studies^(23,24,25,26)^, six months in two^(27,28)^, 12 months in one^(29)^, three months in one^(30)^, and five weeks in one^(31)^.

Participants were school-aged children and adolescents. Twelve studies (52.2%) included fourth-grade students, 13 (56.5%) included fifth-grade students, and 14 (60.9%) included sixth-grade students; several studies enrolled more than one grade level.

Ten studies reported mean participant age, ranging from 8.85 to 13.61 years^(20,24,25,26,28,30,31,32,33,34)^. Across reports that provided age ranges, participants were seven to 17 years old.

Universal interventions predominated (11 studies, 47.8%)^(20,22,23,27,28,29,30,31,35,36,37)^. Eight studies (34.8%) evaluated the KiVa program, which combines universal and targeted components^(6,32,33,34,38,39,40,41)^.

Nineteen studies (82.6%) described interventions with 10 sessions or fewer. Teachers or other school staff delivered at least part of the intervention in 20 studies (87.0%)^(6,20,24,25,26,29,32,33,36,38,39,40,41,42)^.

Eight studies (34.8%) evaluated the multidimensional, school-wide KiVa program^(6,32,33,34,38,39,40,41)^. The remaining 15 studies (65.2%) evaluated more focused approaches: six curricular interventions^(20,22,27,28,29,30)^, five peer-support interventions^(24,25,26,36,37)^, three virtual or computer-based programs^(23,31,35)^, and one support-group intervention that involved bystanders while focusing on victimized students^(42)^.

### Risk of Bias Assessment

Downs and Black scores ranged from 1 to 24 of 28 possible points (median 14.0). Eight studies (34.8%) were classified as low risk of bias, nine (39.1%) as uncertain risk, and six (26.1%) as high risk. Chart 3 presents the reporting, external-validity, internal-bias, confounding, and power scores for each study.

Axford et al.^(38)^ and Desmet et al.^(23)^ had the highest total scores (24 each). The six high-risk studies were Andreou et al.^(27)^, Karna et al.^(32)^, Salmivalli et al.^(6)^, Pack et al.^(36)^, Andreou et al.^(28)^, and Vannini et al.^(31)^.

### Effect of Interventions

The 23 studies contributed to more than one outcome when applicable: 15 assessed defensive behavior, 12 bullying perpetration, 14 bullying victimization, three cyberbullying perpetration, and four cybervictimization. The narrative counts below refer to unique studies, whereas Chart 4 distinguishes unique studies from independent comparisons entered in each meta-analysis.

**Chart 4:** Summary of the nine meta-analyses – Fortaleza, CE, Brazil, 2025.

Synthesis	Outcome and contributing studies	Studies (comparisons)	Participants	Pooled effect (95% CI)	p-value	I2
4A	Defending behavior: asking for help/contacting an adultMerrell^(22)^; Stevens et al.^(29)^	2 (3)	827	MD -0.03 (-0.43 to 0.37)	0.88	84%
4B	Defending/positive behaviorDesmet et al.^(23)^; Menesini et al.^(37)^; Stevens et al.^(29)^	3 (4)	1,103	MD 0.06 (-0.18 to 0.30)	0.61	68%
4C	Bullying perpetration prevalenceAxford et al.^(38)^; Desmet et al.^(23)^; Nocentini and Menesini^(33)^	3 (4)	5,320	RR 0.75 (0.60 to 0.95)	0.02	0%
4D	Cyberbullying perpetration prevalenceDesmet et al.^(23)^; Williford et al.^(40)^	2 (3)	14,101	RR 0.93 (0.61 to 1.40)	0.72	38%
4E	Bullying perpetration, continuous outcome; longer-duration Chaux armChaux et al.^(20)^; Karna et al.^(32)^; Karna et al.^(39)^; Nocentini and Menesini^(33)^	4 (6)	23,699	MD -0.03 (-0.04 to -0.01)	<0.0001	86%
4F	Bullying perpetration, continuous outcome; shorter-duration Chaux armChaux et al.^(20)^; Karna et al.^(32)^; Karna et al.^(39)^; Nocentini and Menesini^(33)^	4 (6)	23,608	MD -0.03 (-0.04 to -0.01)	<0.0001	83%
4G	Bullying victimization prevalenceAxford et al.^(38)^; Desmet et al.^(23)^; Salmivalli et al.^(6)^	3 (3)	9,081	RR 0.63 (0.40 to 0.99)	0.04	0%
4H	Bullying victimization, continuous outcome; shorter-duration Chaux armChaux et al.^(20)^; Karna et al.^(32)^; Karna et al.^(39)^; Nocentini and Menesini^(33)^	4 (6)	23,683	MD -0.05 (-0.08 to -0.02)	0.002	94%
4I	Bullying victimization, continuous outcome; longer-duration Chaux armChaux et al.^(20)^; Karna et al.^(32)^; Karna et al.^(39)^; Nocentini and Menesini^(33)^	4 (6)	23,777	MD -0.06 (-0.09 to -0.03)	0.0003	95%

MD: mean difference; RR: risk ratio; CI: confidence interval; I2: percentage of variability attributable to heterogeneity. Studies were counted once in the study column. Independent, nonoverlapping grade- or school-level strata were entered as separate comparisons. Shorter- and longer-duration intervention arms from Chaux et al.^(20)^ were analyzed in separate syntheses. In Figures 6, 7, 9, and 10, the relevant Chaux et al.^(20)^ duration arm contributes to the pooled estimate; the alternative arm is displayed with zero weight to document the data structure without double counting.

For defensive behavior, seven of 15 studies reported a statistically significant favorable effect^(24,27,29,31,32,34,42)^. Pack et al.^(36)^ described a favorable direction but did not provide a test of statistical significance. Six studies reported no statistically significant effect^(22,23,25,28,35,37)^, and Karna et al.^(39)^ reported mixed findings, including a decrease in defending in one subgroup. Effects varied by assessment timing^(26,27)^, educational level^(29)^, and sample characteristics^(31)^.

Among the 12 studies assessing bullying perpetration, eight reported statistically significant reductions^(20,25,27,32,33,34,39,41)^, whereas four did not^(23,28,37,38)^. Findings varied according to grade or age^(39)^, intervention duration^(20)^, assessment timing^(27)^, and whether outcomes were self- or peer-reported^(32,39)^. Five of the eight studies with favorable findings evaluated KiVa^(32,33,34,39,41)^.

Three studies assessed cyberbullying perpetration^(20,23,40)^. Chaux et al.^(20)^ and Williford et al.^(40)^ reported significant reductions in at least one analysis, with variation by intervention duration or grade group, whereas Desmet et al.^(23)^ found no significant effect.

Among the 14 studies assessing bullying victimization, eight reported statistically significant reductions^(6,30,32,33,34,39,41,42)^ and six did not^(20,23,27,28,37,38)^. In Pfetsch et al.^(30)^, the reduction was evident only with high implementation quality. In van der Ploeg et al.^(42)^, improvement was observed in the short term but was not sustained, and Karna et al.^(39)^ reported age-related variation. Six of the eight studies with favorable findings evaluated KiVa^(6,32,33,34,39,41)^.

Four studies assessed cybervictimization^(6,20,23,40)^. Salmivalli et al.^(6)^ and Williford et al.^(40)^ reported significant favorable findings, whereas Chaux et al.^(20)^ and Desmet et al.^(23)^ reported nonsignificant results.

A descriptive pattern favored multidimensional approaches: seven of the eight KiVa evaluations^(6,32,33,34,38,39,40,41)^ reported a significant favorable effect for at least one outcome, with Axford et al.^(38)^ as the exception. This pattern was not tested in a formal subgroup analysis and therefore should not be interpreted as a comparative effect estimate.

### Quantitative Synthesis of Included Studies: Meta-Analyses

Nine random-effects meta-analyses were conducted after grouping studies by outcome construct, data type, intervention exposure, and assessment timing: two for defensive behavior (4A–4B), four for bullying or cyberbullying perpetration (4C–4F), and three for bullying victimization (4G–4I). Chart 4 reports both unique studies and independent comparisons; the corresponding forest plots are presented in Figures 2–10.

**Figure 2:**
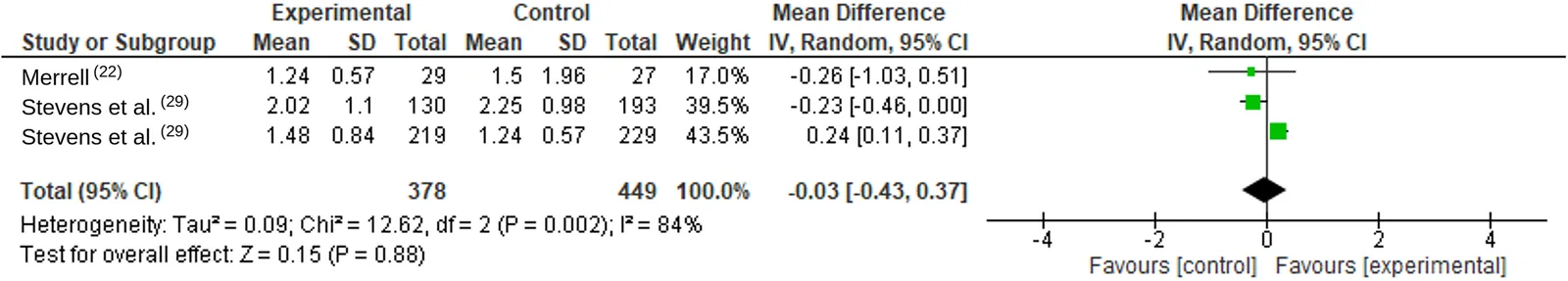
Forest plot of the effect of bystander-focused interventions on defending behavior (asking for help/contacting an adult) – Fortaleza, CE, Brazil, 2025.

**Figure 3:**
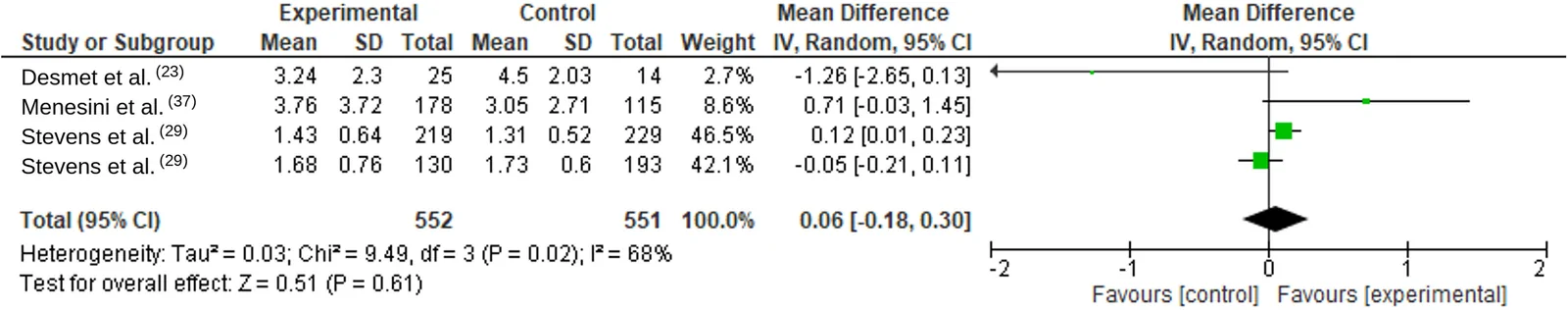
Forest plot of the effect of interventions on defending or positive bystander behavior – Fortaleza, CE, Brazil, 2025.

**Figure 4:**
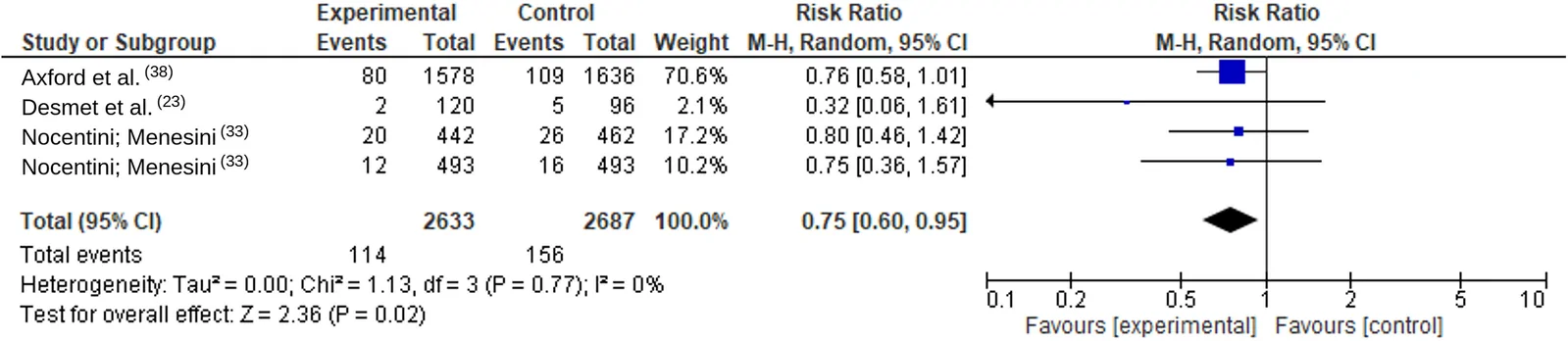
Forest plot of the effect of interventions on bullying perpetration prevalence – Fortaleza, CE, Brazil, 2025.

**Figure 5:**
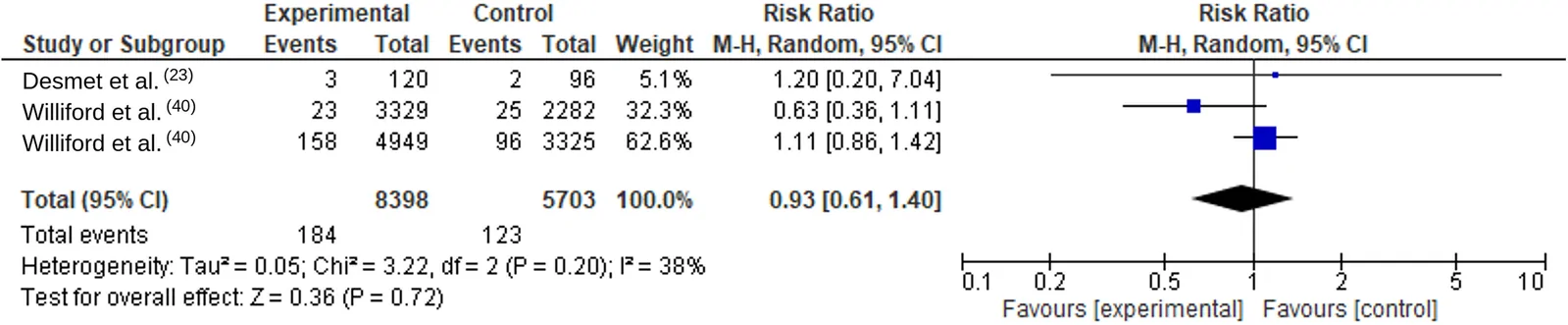
Forest plot of the effect of interventions on cyberbullying perpetration prevalence – Fortaleza, CE, Brazil, 2025.

**Figure 6:**
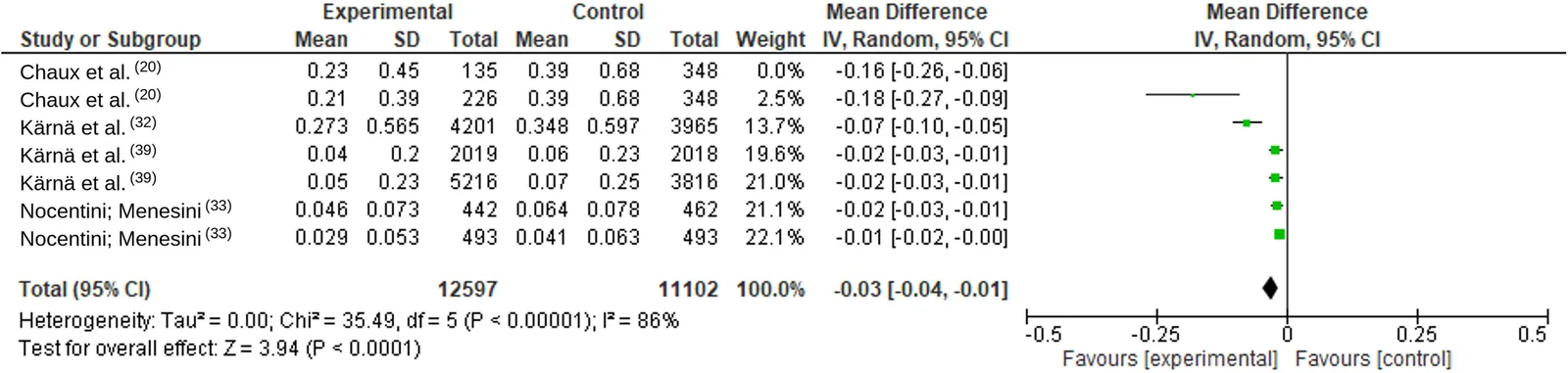
Forest plot of the effect of interventions on bullying perpetration using the longer-duration intervention arm reported by Chaux et al.(20) – Fortaleza, CE, Brazil, 2025.

**Figure 7:**
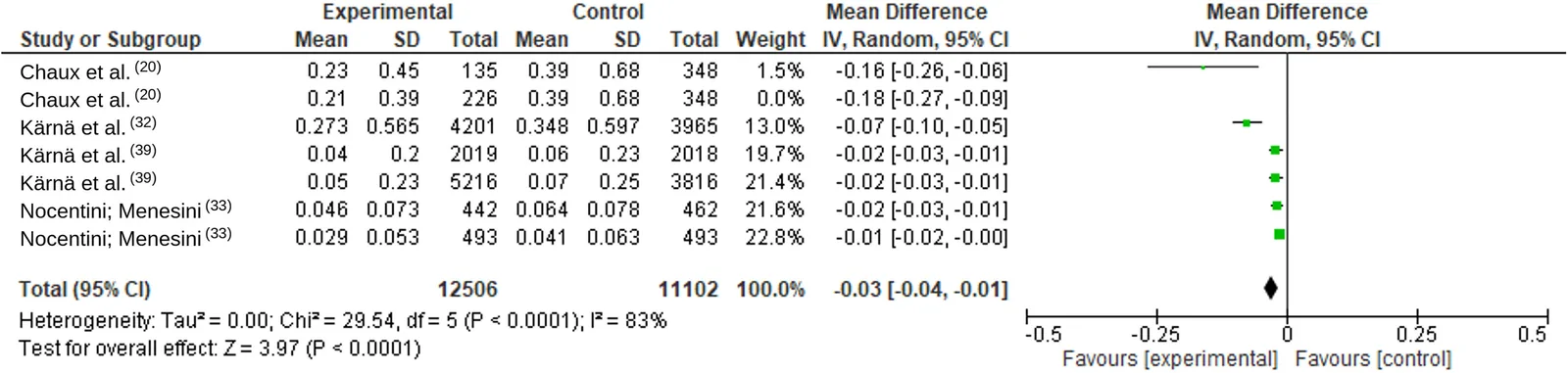
Forest plot of the effect of interventions on bullying perpetration using the shorter-duration intervention arm reported by Chaux et al.(20) – Fortaleza, CE, Brazil, 2025.

**Figure 8:**
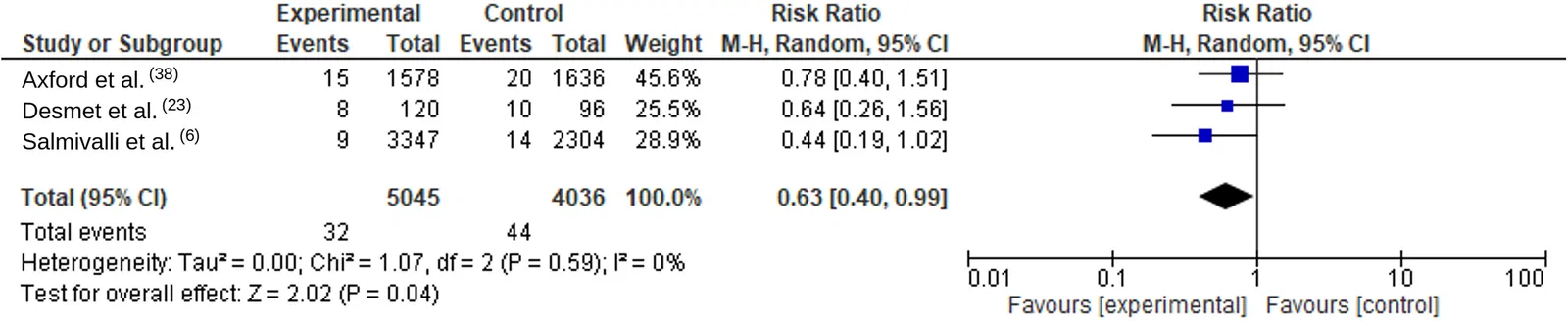
Forest plot of the effect of interventions on bullying victimization prevalence – Fortaleza, CE, Brazil, 2025.

**Figure 9:**
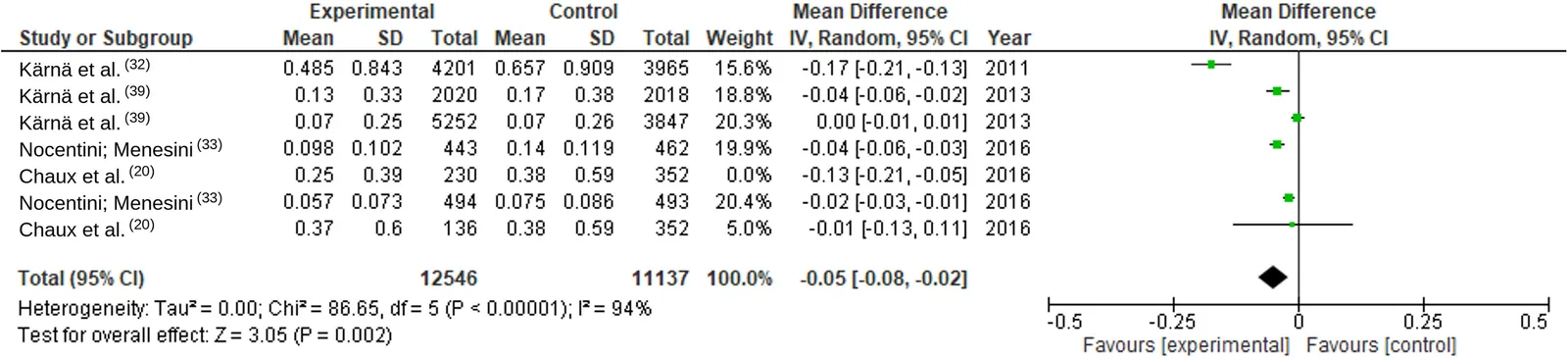
Forest plot of the effect of interventions on bullying victimization using the shorter-duration intervention arm reported by Chaux et al.(20) – Fortaleza, CE, Brazil, 2025.

**Figure 10:**
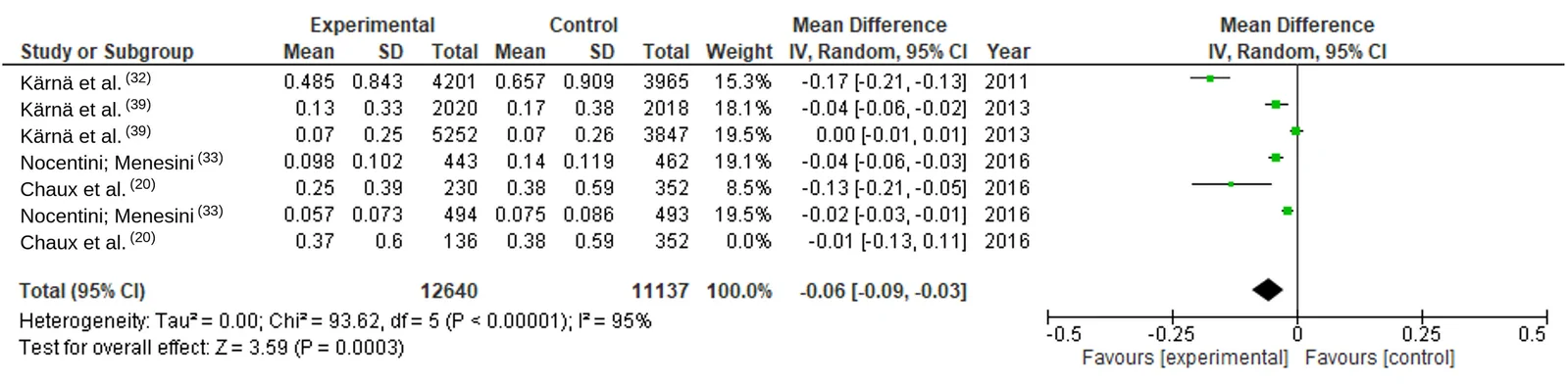
Forest plot of the effect of interventions on bullying victimization using the longer-duration intervention arm reported by Chaux et al.(20) – Fortaleza, CE, Brazil, 2025.

### Meta-Analyses of Defensive Behavior

Synthesis 4A included two studies and three independent educational-level comparisons (827 participants). Bystander-focused interventions did not significantly improve asking for help or contacting an adult (MD −0.03, 95% CI −0.43 to0.37; p = 0.88), and heterogeneity was high (I2 = 84%). The corresponding forest plot is presented in Figure 2.

Synthesis 4B included three studies and four comparisons (1,103 participants). The pooled effect on defending or positive bystander behavior was not significant (MD 0.06, 95% CI −0.18 to 0.30; p = 0.61), with moderate heterogeneity (I2 = 68%). The corresponding forest plot is presented in Figure 3.

Stevens et al.^(29)^ contributed separate, nonoverlapping primary- and secondary-school comparisons to syntheses 4A and 4B; the study itself was counted once in the number of unique studies.

### Meta-Analyses of Bullying and Cyberbullying Perpetration

Synthesis 4C included three studies and four comparisons (5,320 participants). Bystander-focused interventions reduced bullying perpetration prevalence (RR 0.75, 95% CI 0.60 to 0.95; p = 0.02), with no observed statistical heterogeneity (I2 = 0%). The corresponding forest plot is presented in Figure 4.

Synthesis 4D included two studies and three grade-group comparisons (14,101 participants). The pooled effect on cyberbullying perpetration was not significant (RR 0.93, 95% CI 0.61 to 1.40; p = 0.72), and heterogeneity was low (I2 = 38%). The corresponding forest plot is presented in Figure 5.

Williford et al.^(40)^ contributed separate data for fourth- to sixth-grade and eighth- to ninth-grade students to synthesis 4D.

Synthesis 4E combined four studies and six comparisons (23,699 participants), using the longer-duration intervention arm reported by Chaux et al.^(20)^. Bullying perpetration scores were lower in the intervention groups (MD −0.03, 95% CI −0.04 to -0.01; p < 0.0001), with high heterogeneity (I2 = 86%). The corresponding forest plot is presented in Figure 6.

Synthesis 4F included the same four studies and six comparisons but used the shorter-duration Chaux arm (23,608 participants). The pooled effect was MD −0.03 (95% CI −0.04 to −0.01; p < 0.0001), also with high heterogeneity (I2 = 83%). The corresponding forest plot is presented in Figure 7.

Karna et al.^(39)^ and Nocentini and Menesini^(33)^ contributed independent grade- or school-level strata to syntheses 4C, 4E, and 4F. These strata were treated as separate comparisons without counting the corresponding publication as more than one study.

### Meta-Analyses of Bullying Victimization

Synthesis 4G included Axford et al.^(38)^, Desmet et al.^(23)^, and Salmivalli et al.^(6)^ (9,081 participants). Interventions reduced bullying victimization prevalence (RR 0.63, 95% CI 0.40 to 0.99; p = 0.04), with no observed heterogeneity (I2 = 0%). The corresponding forest plot is presented in Figure 8.

Synthesis 4H included four studies and six comparisons (23,683 participants) and used the shorter-duration intervention arm from Chaux et al.^(20)^. Bullying victimization scores were lower in the intervention groups (MD −0.05, 95% CI −0.08 to −0.02; p = 0.002), with high heterogeneity (I2 = 94%). The corresponding forest plot is presented in Figure 9.

Synthesis 4I included the same four studies and six comparisons, using the longer-duration Chaux arm (23,777 participants). The pooled effect was MD −0.06 (95% CI −0.09 to −0.03; p = 0.0003), with high heterogeneity (I2 = 95%). The corresponding forest plot is presented in Figure 10.

Karna et al.^(39)^ and Nocentini and Menesini^(33)^ contributed independent educational-level strata to syntheses 4H and 4I. The alternative Chaux duration arm was excluded from each respective pooled estimate to prevent double counting.

Across syntheses, evidence supported reductions in traditional bullying perpetration and victimization. Pooled effects were not significant for cyberbullying perpetration or defensive behavior. Continuous-outcome syntheses showed moderate to high heterogeneity (I2 = 68%–95%), and the small number of studies precluded reliable formal exploration of its causes.

### Evidence Certainty Ranking

GRADE certainty was high for bullying perpetration prevalence (RR 0.75) and bullying victimization prevalence (RR 0.63). Certainty was very low for the seven remaining syntheses because of combinations of study limitations, inconsistency, and imprecision.

Chart 5 presents the complete certainty judgment for each meta-analysis. Reporting bias was not formally tested because every synthesis contained fewer than 10 unique studies; no certainty rating was downgraded solely on the basis of an uninterpretable funnel plot.

**Chart 5:** GRADE assessment of certainty for the nine meta-analyses – Fortaleza, CE, Brazil, 2025.

Outcome (synthesis)	Studies (comparisons)	Participants	Effect (95% CI)	Reasons for downgrading/judgment	Certainty
4A Defending: ask for help/contact an adult	2 (3)	827	MD -0.03 (-0.43 to 0.37)	Study limitations; very serious inconsistency (I2 = 84%); serious imprecision	Very low
4B Defending/positive behavior	3 (4)	1,103	MD 0.06 (-0.18 to 0.30)	Study limitations; serious inconsistency (I2 = 68%); serious imprecision	Very low
4C Bullying perpetration prevalence	3 (4)	5,320	RR 0.75 (0.60 to 0.95)	No serious concerns identified in the domains evaluated	High
4D Cyberbullying perpetration prevalence	2 (3)	14,101	RR 0.93 (0.61 to 1.40)	Study limitations; inconsistency; serious imprecision	Very low
4E Bullying perpetration, longer-duration arm	4 (6)	23,699	MD -0.03 (-0.04 to -0.01)	Study limitations; very serious inconsistency (I2 = 86%)	Very low
4F Bullying perpetration, shorter-duration arm	4 (6)	23,608	MD -0.03 (-0.04 to -0.01)	Study limitations; very serious inconsistency (I2 = 83%)	Very low
4G Bullying victimization prevalence	3 (3)	9,081	RR 0.63 (0.40 to 0.99)	No serious concerns identified in the domains evaluated	High
4H Bullying victimization, shorter-duration arm	4 (6)	23,683	MD -0.05 (-0.08 to -0.02)	Study limitations; very serious inconsistency (I2 = 94%)	Very low
4I Bullying victimization, longer-duration arm	4 (6)	23,777	MD -0.06 (-0.09 to -0.03)	Study limitations; very serious inconsistency (I2 = 95%)	Very low

CI: confidence interval; MD: mean difference; RR: risk ratio; I2: heterogeneity. Independent, nonoverlapping strata were counted as comparisons. Formal small-study-effect tests were not conducted because each synthesis contained fewer than 10 unique studies.

## DISCUSSION

This review found that school-based interventions incorporating bystander strategies were associated with reductions in traditional bullying perpetration and victimization. In contrast, pooled estimates did not show significant effects on cyberbullying perpetration or defensive behavior. Because several continuous-outcome syntheses were highly heterogeneous, those estimates should be interpreted cautiously.

Seven of eight KiVa evaluations reported a favorable effect for at least one outcome, suggesting that multidimensional, whole-school programs may offer a useful context for bystander components^(6,32,33,34,39,40,41)^. However, this was a descriptive pattern rather than a formal between-program comparison. The review did not conduct a subgroup analysis, and differences in participants, implementation, outcome measurement, and follow-up could explain the observed pattern.

The two prevalence meta-analyses indicated reductions in self-reported traditional bullying perpetration and victimization and were rated as high-certainty evidence. The certainty of all other syntheses was very low. Therefore, the significant continuous-outcome estimates for perpetration and victimization should not be interpreted as equally robust, particularly because I2 ranged from 83% to 95% in those analyses.

A previous meta-analysis of 12 studies and 12,784 participants reported a modest increase in defensive bystander behavior (Hedges g = 0.20, 95% CI 0.11 to 0.29; p < 0.001), with larger effects among older students^(12)^. Differences from the present findings may reflect the earlier review period, broader intervention inclusion, and different definitions and measures of defending behavior.

Defending is a complex behavior influenced by individual, peer, family, classroom, and school factors^(43,44)^. An intervention may reduce reinforcement of bullying or increase help-seeking without producing a measurable increase in direct defending. This may explain why reductions in perpetration and victimization were observed even when pooled defending outcomes were nonsignificant.

The differential pattern across outcomes suggests that bystander components may operate less through increases in direct defending and more through changes in peer norms, reduced social reinforcement of aggression, and greater use of safe help-seeking and adult support. This interpretation is consistent with the favorable descriptive pattern observed in multidimensional programs, although comparative effectiveness was not tested. Accordingly, implementation should combine student education with staff preparation, institutional response protocols, and monitoring of school climate. School nurses and other health professionals can contribute by identifying bullying-related psychological and somatic consequences, supporting affected students and families, and coordinating referral pathways with education teams.

The absence of a significant pooled cyberbullying effect may reflect the small number of eligible studies and limited attention to characteristics specific to online contexts, including anonymity, rapid dissemination, and the persistence of digital content. The wide confidence interval for cyberbullying perpetration was compatible with both benefit and no benefit, so the result should be viewed as inconclusive rather than evidence of ineffectiveness.

For practice and policy, bystander education can be incorporated into comprehensive school antibullying programs, but implementation should include staff training, monitoring of fidelity, clear referral pathways, and outcome evaluation. Cyberbullying content and safe forms of help-seeking should be addressed explicitly rather than assumed to be just transferred from traditional bullying programs.

Strengths of this review include broad database and gray-literature searching, no language or date restriction, independent review procedures, and explicit certainty assessment. Important limitations remain. Six studies were classified as high risk of bias and nine as uncertain risk; these limitations may exaggerate or attenuate effects and contributed to downgrading several outcomes. Intervention formats and outcome measures varied substantially, and the small number of studies in each synthesis prevented formal subgroup, meta-regression, sensitivity, or reliable publication-bias analyses. Multiple reports also contributed independent grade-level strata, so participant totals across meta-analyses must not be summed as a count of unique individuals. Most studies were conducted in settings with high Human Development Index, which may limit transferability to other contexts.

## CONCLUSION

School-based interventions that include bystander-focused strategies may reduce traditional bullying perpetration and victimization, with high-certainty evidence for the two prevalence outcomes. Evidence for defensive behavior and cyberbullying remains very uncertain, and continuous-outcome estimates were affected by substantial heterogeneity.

Schools and health and education policymakers should integrate bystander components within comprehensive programs while monitoring implementation and safety. Future studies should use adequately powered controlled designs, standardized outcome definitions, prespecified analyses, longer follow-up, and samples from diverse socioeconomic and cultural settings, with explicit evaluation of cyberbullying and different forms of defending.

## Data Availability

The data extracted for this review and the forest plots supporting the meta-analyses are provided within this article.
