# Supplementary material for: Effects of bystander interventions on school bullying: a systematic review and meta-analysis
Source: Rev Esc Enferm USP. 2026 Nov 2;60:e20260029. doi: 10.1590/1980-220X-REEUSP-2026-0029en (PMC13739456; doi:10.1590/1980-220X-REEUSP-2026-0029en)
Supplement: Translation

# Translation: Efectos de las intervenciones de los testigos presenciales en el acoso escolar: revisión sistemática y metaanálisis

**DOI:** 10.1590/1980-220X-REEUSP-2026-0029es

**Authors:** Luiza Luana de Araújo Lira Bezerra, Raquel Sampaio Florêncio, Dayse Lorrane Gonçalves Alves, Valter Cordeiro Barbosa, Thereza Maria Magalhães Moreira, Edna Maria Camelo Chaves, Mirna Albuquerque Frota, Bruna Rodrigues Nunes, Thiago Santos Garces, Açucena Leal de Araújo, Ilvana Lima Verde Gomes

**Affiliations:** 1 Universidade Estadual do Ceará, Centro de Ciências da Saúde, Programa de Pós-Graduação em Saúde Coletiva, Fortaleza, CE, Brasil.; 2 Universidade Estadual do Ceará, Centro de Ciências da Saúde, Programa de Pós-Graduação Cuidados Clínicos em Enfermagem e Saúde, Fortaleza, CE, Brasil.; 3 Universidade de Fortaleza, Programa de Pós-graduação em Saúde Coletiva, Fortaleza, CE, Brasil.; 4 Universidade Regional do Cariri, Centro de Ciências da Saúde, Departamento de Enfermagem, Crato, CE, Brasil.

**Keywords:** Acoso Escolar, Ciberacoso, Instituciones Académicas, Revisión Sistemática

## Abstract

**Objetivo:**

Sintetizar la evidencia sobre la efectividad de las intervenciones escolares dirigidas a los testigos para reducir el acoso escolar y el ciberacoso entre niños y adolescentes.

**Método:**

Esta revisión sistemática y metaanálisis siguió las directrices de PRISMA 2020 y se registró en PROSPERO (CRD42021244706). Se identificaron estudios controlados experimentales y cuasi-experimentales mediante bases de datos, literatura gris, listas de referencias y revisiones. Revisores independientes seleccionaron los estudios, extrajeron los datos y evaluaron la calidad utilizando la lista de verificación de Downs y Black. Los metaanálisis de efectos aleatorios estimaron las razones de riesgo (RR) y las diferencias medias; la certeza se evaluó con el método GRADE.

**Resultados:**

Se incluyeron veintitrés estudios, con muestras que oscilaron entre 54 y 23 430 participantes. Las intervenciones redujeron la victimización por acoso escolar tradicional (RR 0,63; IC del 95 %: 0,40–0,99; p = 0,04; I2 = 0 %) y la perpetración (RR 0,75; IC del 95 %: 0,60–0,95; p = 0,02; I2 = 0 %). Los efectos no fueron significativos en cuanto a la perpetración del acoso cibernético ni al comportamiento defensivo de los espectadores. El nivel de certeza fue alto para los resultados relacionados con el acoso tradicional y muy bajo para el resto de los resultados.

**Conclusión:**

Las intervenciones que incluyen un componente dirigido a los espectadores pueden reducir la perpetración y la victimización por acoso tradicional, pero la evidencia sobre el acoso cibernético y el comportamiento defensivo sigue siendo limitada.

## INTRODUCCIÓN

El acoso escolar, un problema de salud pública muy frecuente, afecta profundamente la salud mental y física, aumentando el riesgo de ansiedad, depresión e ideas suicidas, con efectos que pueden persistir hasta la edad adulta. En consecuencia, la prevención del acoso escolar es una prioridad en las políticas de salud y educación.

Si bien las intervenciones escolares contra el acoso escolar muestran efectos de pequeños a moderados en la reducción de la perpetración y la victimización, su eficacia a largo plazo sigue sin estar clara^(1,2,3,4)^. Tradicionalmente, estas intervenciones se centran en las víctimas y los agresores y pueden restar importancia al papel de los espectadores^(5)^. Sin embargo, el acoso escolar es un fenómeno grupal, y los espectadores pueden influir en su dinámica^(6)^. La observación pasiva puede reforzar el acoso escolar, mientras que la intervención activa puede ayudar a interrumpirlo.

A pesar de la evidencia que destaca la importancia de las intervenciones de los espectadores^(7,8,9,10)^, las investigaciones que evalúan directamente su impacto en la prevalencia del acoso escolar siguen siendo limitadas^(11,12,13)^. Un metaanálisis de 12 estudios (12 784 participantes) encontró efectos positivos modestos (g de Hedges = 0,20, IC del 95 %: 0,11–0,29, p-valor < 0,001) de las intervenciones contra el acoso escolar en el aumento del comportamiento defensivo de los espectadores, con efectos más marcados en los estudiantes de preparatoria (ES = 0,43) que en los estudiantes más jóvenes (ES = 0,14, p-valor < 0,05). Sin embargo, esta revisión, limitada a estudios publicados entre 1998 y 2010, se centró en el comportamiento defensivo y la empatía, en lugar de evaluar directamente la reducción de los casos de acoso escolar o ciberacoso. Dado el reconocido papel de los testigos en la prevención, esta revisión sistemática tiene como objetivo sintetizar la evidencia actual sobre la efectividad de las intervenciones escolares dirigidas a los testigos para reducir el acoso escolar.

## MÉTODO

### Protocolo y Registro

Esta revisión sistemática se informó de acuerdo con los Elementos Preferidos para el Informe de Revisiones Sistemáticas y Metaanálisis (PRISMA 2020)^(14)^ y se llevó a cabo siguiendo las directrices metodológicas de Cochrane^(15)^. El protocolo se registró en el Registro Internacional Prospectivo de Revisiones Sistemáticas (PROSPERO; CRD42021244706).

### Pregunta de Investigación y Criterios de Elegibilidad

El marco PICO^(16,17)^ sirvió de guía para la pregunta: «¿Cuál es el efecto de las intervenciones escolares dirigidas a los espectadores sobre el acoso escolar?» Los informes elegibles evaluaban programas escolares centrados en la participación de los espectadores en el acoso escolar o el ciberacoso entre niños y adolescentes de cualquier género; utilizaban un diseño controlado experimental o cuasi-experimental; y reportaban al menos un resultado preespecificado: intervención o comportamiento de defensa por parte de los espectadores, victimización por acoso escolar, perpetración de acoso escolar, perpetración de ciberacoso o cibervictimización. Se consideraron elegibles los artículos revisados por pares, capítulos de libros, tesis y disertaciones, sin restricciones de fecha ni de idioma. Para la síntesis, los estudios se agruparon por constructo de resultado, tipo de datos (dicotómicos o continuos), exposición a la intervención, momento de la evaluación y disponibilidad de datos de grupos clínicamente comparables.

### Fuentes de Información

La búsqueda se actualizó por última vez en junio de 2025 en PubMed, Web of Science, Scopus, SciELO, Embase, LILACS, CINAHL, ERIC, el Registro Central Cochrane de Ensayos Controlados, ScienceDirect y PsycArticles. Las fuentes de literatura gris incluyeron la Biblioteca Digital Brasileña de Tesis y Disertaciones, el Catálogo de Tesis y Disertaciones de la CAPES, ProQuest Dissertations and Theses y Google Scholar. También se revisaron manualmente las listas de referencias de los estudios incluidos y de las revisiones sistemáticas y metaanálisis relevantes.

### Estrategias de Búsqueda

La estrategia principal (S1) combinó vocabularios controlados (DeCS, MeSH y Emtree) y términos de texto libre organizados según el modelo PICOS, sin un bloque de búsqueda de comparadores. Dado que la estrategia completa no era compatible con ERIC, ScienceDirect, LILACS, SciELO, Google Scholar, ProQuest Dissertations and Theses, el catálogo de la CAPES y PsycArticles, en esas fuentes se utilizaron combinaciones simplificadas de palabras clave (A1–A4) desarrolladas con el apoyo de bibliotecarios. Los cuadros 1 y 2 presentan las estrategias completas.

**Cuadro 1:** Estrategia de búsqueda (S1) por grupos y descriptores (PICOS) – Fortaleza, CE, Brasil, 2025.

Grupo de descriptores	Descriptores
Población	(child OR children OR childhood OR boy OR girl OR “school child” OR “school boy” OR “school children” OR “school girl” OR “school-going” OR schoolboy OR schoolchild OR schoolchildren OR schoolgirl OR schoolgirls OR schoolgoing OR adolescent OR adolescence OR teen OR teenager OR youth OR student OR “elementary student” OR “elementary pupils” OR “elementary school pupil” OR “elementary school student” OR “high school student” OR “middle school student” OR school OR “educational institution” OR “primary school” OR “secondary school” OR “high school” OR “middle school” OR “elementary school” OR “school-aged”)
Intervención	(witness OR “witness corroboration” OR “eye witness” OR eyewitness OR testimony OR “bullying witnessed” OR witnesses OR witnessed OR “witnessing bullying” OR “bullying witnessed” OR “bullying witnessing” OR bystander OR spectator)
Comparación	Control/usual practice/no intervention; evaluado durante la fase de elegibilidad y no utilizado como término de búsqueda
Resultados	(bullying OR cyberbullying OR “cyber-bullying” OR “e-bullying” OR “electronic bullying” OR “internet bullying” OR “online bullying” OR aggression OR “aggressive attitude” OR “aggressive behavior” OR “aggressive behaviour” OR “aggressive reaction” OR aggressiveness OR aggressivity OR “counter aggression” OR counteraggression OR provocation OR hostility OR “hostile verb” OR “psychological antagonism” OR threat OR “school bullying” OR “school victimization” OR “victimization among school” OR “bully victimization” OR “school bullying victimization” OR “bully-victim” OR “peer victimization” OR “peer abuse” OR “psychological victimization” OR “stigma experienced” OR “involvement in bullying” OR “peer harassment” OR “relational aggression” OR “peer rejection” OR “online harassment” OR “cyber-harassment” OR “cyberharassment” OR “electronic harassment” OR “internet harassment”)
Diseño del estudio	(“Intervention Study” OR “intervention studies” OR “intervention trial” OR “interventional studies” OR “interventional study” OR “interventional trial” OR “quasi-randomised controlled trials” OR “controlled before-and-after” OR “Controlled Before After Studies” OR “Controlled Before-After Study” OR “CBA Studies” OR “Controlled Before and After Studies” OR “randomized controlled trial” OR “Randomized Controlled Trials as Topic” OR “randomised controlled study” OR “randomized controlled study” OR “controlled clinical trial” OR “controlled clinical comparison” OR “controlled clinical drug trial” OR “controlled clinical experiment” OR “controlled clinical study” OR “controlled clinical test” OR “Controlled Clinical Trials as Topic” OR “randomized controlled trials” OR “randomized controlled trial” OR “pragmatic clinical trials as topic” OR “randomized controlled trials” OR “randomized controlled trials as topic” OR “randomized controlled trial as topic” OR “Random Allocation” OR “Randomization” OR “random allocation” OR “randomisation” OR “double blind procedure” OR “double-blind method” OR “double blind clinical trial” OR “double blind comparison” OR “double blind design” OR “double blind studies” OR “double blind study” OR “double blind test” OR “double blind trial” OR “double masked clinical study” OR “double masked clinical trial” OR “double masked comparison” OR “double masked design” OR “double masked method” OR “double masked procedure” OR “double masked study” OR “double masked test” OR “double masked trial” OR “double-blind clinical study” OR “double blind method” OR “Double-Blind Methods” OR “Single-Blind Method” OR “single blind procedure” OR “single blind clinical study” OR “single blind clinical trial” OR “single blind comparison” OR “single blind design” OR “single blind studies” OR “single blind study” OR “single blind test” OR “single blind trial” OR “single masked clinical study” OR “single masked clinical trial” OR “single masked comparison” OR “single masked design” OR “single masked method” OR “single masked procedure” OR “single masked study” OR “single masked studies” OR “single masked test” OR “single masked trial” OR “single-blind method” OR “comparative study” OR “comparative studies” OR comparison OR “Evaluation Studies as Topic” OR evaluation OR “evaluation studies” OR “evaluation study” OR “Evaluation Report” OR “Evaluation Reports” OR Critique OR “Critiques” OR “Evaluation Research” OR “Use-Effectiveness” OR “Use Effectiveness” OR “Pre-Post Tests” OR “Pre Post Tests” OR “Qualitative Evaluation” OR “Qualitative Evaluations” OR “Quantitative Evaluation” OR “Quantitative Evaluations” OR “Theoretical Effectiveness” OR “Evaluation Indexes” OR “Evaluation Methodology” OR “Evaluation Methodologies” OR “Prospective Studies” OR “Prospective Study” OR “prospective method”)

**Cuadro 2:** Estrategias de búsqueda A1, A2, A3 y A4 por palabras clave – Fortaleza, CE, Brasil, 2025.

Bases de datos		Estrategia con palabras clave
ScienceDirect	A1	school AND (child OR adolescent) AND bullying AND (witness OR bystander OR testimony) AND (“Intervention Study” OR “randomized controlled trial”)
LILACS/Scielo/CTD-CAPES	A2	(school OR escola OR escuela) AND (child OR children OR criança OR niño OR adolescent OR adolescente) AND (bullying OR acoso)
ERIC/ProQuest/PsycArticles	A3	school AND (child OR adolescent) AND bullying AND (witness OR bystander OR testimony)
Google Scholar	A4	school AND (child OR adolescent) AND bullying AND (witness OR bystander OR testimony) AND (“randomized clinical trial” OR “clinical study”)

### Selección de Estudios y Extracción de Datos

Dos revisores examinaron de manera independiente los títulos y los resúmenes y, posteriormente, evaluaron los textos completos de los informes potencialmente elegibles. Las discrepancias fueron resueltas por un tercer revisor. Los registros duplicados se eliminaron electrónicamente y se verificaron manualmente; ninguna herramienta automatizada tomó decisiones sobre la elegibilidad. Se utilizaron hojas de cálculo de Microsoft Excel para gestionar los registros y extraer los datos. Tres revisores recolectaron información bibliográfica (autores, año y país), el diseño del estudio, el tamaño de la muestra y las características de los participantes, las características de la intervención y del comparador, el personal encargado de la implementación, el número y la duración de las sesiones, el seguimiento, las definiciones de los resultados y los instrumentos de medición, las estadísticas resumidas a nivel de grupo, las estimaciones de efecto y la información necesaria para la evaluación del riesgo de sesgo. La información poco clara se verificó con el informe completo y las publicaciones relacionadas. No se imputaron valores de resultados; los datos se agruparon únicamente cuando las estadísticas reportadas eran directamente utilizables o podían derivarse de los datos grupales reportados. De lo contrario, los hallazgos se mantuvieron en la síntesis narrativa.

### Evaluación del Riesgo de Sesgo

La calidad metodológica se evaluó con la lista de verificación de Downs y Black de 27 ítems en cinco dominios^(18)^. Se seleccionó este instrumento porque la revisión incluía tanto estudios de intervención controlados aleatorizados como no aleatorizados, mientras que el RoB 2 está diseñado específicamente para ensayos aleatorizados y no proporcionaría un marco único común para todos los diseños elegibles. El ítem 27 (potencia estadística) se dicotomizó como 1 para los estudios que reportaban al menos un 80 % de potencia y 0 en los demás casos. Las puntuaciones totales oscilaron entre 0 y 28 y se clasificaron como riesgo alto (0–5), riesgo incierto (6–18) o riesgo bajo (19–28)^(19)^. Dos revisores evaluaron cada estudio de manera independiente, y un tercer revisor resolvió las discrepancias. Las puntuaciones por dominio y la clasificación de cada estudio incluido se presentan en el Cuadro 3.

**Cuadro 3:** Puntuaciones por dominios de Downs y Black y clasificación del riesgo de sesgo para cada estudio incluido – Fortaleza, CE, Brasil, 2025.

Estudio	Informes (0–11)	Validez externa (0–3)	Sesgo interno (0–7)	Factores de confusión (0–6)	Poder (0–1)	Total (0–28)	Clasificación
Axford et al.^(38)^	11	1	6	5	1	24	Baja
Chaux et al.^(20)^	9	1	4	5	0	19	Baja
Desmet et al.^(23)^	11	2	5	5	1	24	Baja
Kolic-Vehovec et al.^(35)^	9	1	5	4	0	19	Baja
Karna et al.^(39)^	8	3	4	2	0	17	Incierta
Menesini et al.^(37)^	5	2	3	1	1	12	Incierta
Midgett et al.^(24)^	6	2	2	2	1	13	Incierta
Midgett et al.^(25)^	9	2	4	3	0	18	Incierta
Andreou et al.^(27)^	3	0	1	0	1	5	Alta
Karna et al.^(32)^	4	0	1	0	0	5	Alta
Stevens et al.^(29)^	9	3	5	5	0	22	Baja
Williford et al.^(40)^	9	3	5	5	0	22	Baja
Salmivalli et al.^(6)^	4	0	0	0	0	4	Alta
Midgett et al.^(26)^	6	1	4	3	0	14	Incierta
Pack et al.^(36)^	1	0	0	0	0	1	Alta
Pfetsch et al.^(30)^	7	0	1	0	1	9	Incierta
Saarento et al.^(34)^	8	3	4	4	0	19	Baja
van der Ploeg et al.^(42)^	4	0	1	0	1	6	Incierta
Yang and Salmivalli^(41)^	9	3	4	4	0	20	Baja
Andreou et al.^(28)^	4	0	1	0	0	5	Alta
Nocentini and Menesini^(33)^	9	0	2	2	1	14	Incierta
Vannini et al.^(31)^	4	0	1	0	0	5	Alta
Merrell^(22)^	6	1	4	3	0	14	Incierta

Clasificación: 0–5, riesgo alto; 6–18, riesgo incierto; 19–28, riesgo bajo. Midgett et al.^(25)^ se refiere al estudio sobre compañeros-defensores; Midgett et al.^(26)^ se refiere al estudio sobre estudiantes de secundaria.

### Síntesis de Datos

Se preparó una síntesis descriptiva para todos los estudios incluidos. Se llevó a cabo un metanálisis de efectos aleatorios cuando los estudios eran suficientemente comparables en cuanto a población, intervención, constructo de resultado, escala de medición y momento de evaluación. Se dio prioridad a la primera evaluación posterior a la intervención, ya que los esquemas de seguimiento eran escasos y heterogéneos. Cada estudio se contó una sola vez en las descripciones de la base de evidencia, mientras que los estratos independientes y no superpuestos a nivel de grado o escuela podían aportar comparaciones separadas a un metanálisis. Cuando Chaux et al.^(20)^ informaron sobre grupos de intervención de diferentes duraciones, los grupos de menor y mayor duración se evaluaron en síntesis separadas para que los participantes no se contaran dos veces dentro de la misma estimación combinada.

Se utilizaron razones de riesgo (RR) con intervalos de confianza (IC) del 95 % para los resultados dicotómicos, y diferencias medias (DM) con IC del 95 % para los resultados continuos. Los datos dicotómicos se agruparon con el método de Mantel-Haenszel y los datos continuos con métodos de varianza inversa en Review Manager, utilizando modelos de efectos aleatorios.

La heterogeneidad estadística se evaluó con la Q de Cochran, la prueba de chi-cuadrado y el índice I2. Los valores de I2 inferiores al 50 % se interpretaron como heterogeneidad baja, entre el 50 % y el 75 % como moderada, y superiores al 75 % como alta. Se examinaron posibles fuentes clínicas comparando las poblaciones, los formatos y la duración de las intervenciones, las definiciones de los resultados, los instrumentos de medición, los niveles educativos y el momento de la evaluación en los distintos estudios. No se realizaron análisis formales de subgrupos, meta regresión ni análisis de sensibilidad, ya que cada síntesis contenía pocos estudios y las mediciones de los resultados eran muy variables.

El riesgo de sesgo debido a la falta de resultados se consideró cualitativamente mediante la comparación de los métodos y resultados reportados y el examen de la integridad de los resultados en los distintos informes. Dado que cada metaanálisis contenía menos de 10 estudios únicos, no se interpretaron los gráficos de embudo ni las pruebas estadísticas para detectar efectos de estudios pequeños, ya que habrían tenido una potencia muy baja.

### Clasificación de la Certeza de la Evidencia

Se utilizó el enfoque GRADE para calificar la certeza de cada síntesis de acuerdo con el riesgo de sesgo, la inconsistencia, la indirectidad, la imprecisión y otras consideraciones relevantes^(21)^. La certeza se clasificó como alta, moderada, baja o muy baja. Dos revisores realizaron la evaluación de manera independiente y resolvieron las diferencias por consenso. Los juicios finales se presentan en la sección Resultados.

## RESULTADOS

Las búsquedas electrónicas identificaron 3,134 registros (2,866 de bases de datos bibliográficas y 268 de otras fuentes electrónicas). Tras eliminar 516 duplicados, se evaluaron 2,618 registros y se excluyeron 2,535, lo que dejó 83 informes para la evaluación del texto completo. Se excluyeron 68 informes de texto completo y 15 cumplieron con los criterios de elegibilidad. Las búsquedas en revisiones sistemáticas anteriores y en listas de referencias identificaron 780 registros adicionales: 703 fueron excluidos tras la selección de títulos y resúmenes, 16 informes no pudieron recuperarse y 61 fueron evaluados en su totalidad. Se excluyeron 53, incluidos 13 informes ya identificados mediante la búsqueda electrónica, lo que dio como resultado ocho informes elegibles adicionales. En total, se incluyeron 23 estudios representados por 23 informes (22 artículos de revistas y una tesis) (Figura 1).

**Figura 1:** Flujograma PRISMA 2020 de la selección de estudios – Fortaleza, CE, Brasil, 2025.

### Aspectos de los Estudios Incluidos

Los 23 estudios incluidos se publicaron entre 2000 y 2020. Finlandia aportó seis estudios (26,1 %) y Estados Unidos, cinco (21,7 %). Todos los informes se publicaron en inglés, y 22 (95,7 %) eran artículos de revista. Diecisiete estudios (73,9 %) utilizaron diseños controlados aleatorizados, y las muestras de los estudios individuales oscilaron entre 54 y 23 430 participantes.

La duración de la intervención osciló entre uno y 20 meses; dos estudios no informaron la duración^(20,22)^. Catorce estudios (60,9 %) no informaron sobre el seguimiento. Entre los nueve estudios con seguimiento, la evaluación se realizó al mes en cuatro estudios^(23,24,25,26)^, a los seis meses en dos^(27,28)^, a los 12 meses en uno^(29)^, a los tres meses en uno^(30)^ y a las cinco semanas en uno^(31)^.

Los participantes eran niños y adolescentes en edad escolar. Doce estudios (52,2 %) incluyeron a estudiantes de cuarto grado, 13 (56,5 %) incluyeron a estudiantes de quinto grado y 14 (60,9 %) incluyeron a estudiantes de sexto grado; varios estudios incluyeron a participantes de más de un nivel de grado.

Diez estudios informaron la edad promedio de los participantes, que osciló entre 8,85 y 13,61 años^(20,24,25,26,28,30,31,32,33,34)^. En los informes que proporcionaron rangos de edad, los participantes tenían entre siete y 17 años.

Predominaron las intervenciones universales (11 estudios, 47,8 %)^(20,22,23,27,28,29,30,31,35,36,37)^. Ocho estudios (34,8 %) evaluaron el programa KiVa, que combina componentes universales y específicos^(6,32,33,34,38,39,40,41)^.

Diecinueve estudios (82,6 %) describieron intervenciones de 10 sesiones o menos. Los maestros u otro personal escolar impartieron al menos parte de la intervención en 20 estudios (87,0 %)^(6,20,24,25,26,29,32,33,36,38,39,40,41,42)^.

Ocho estudios (34,8 %) evaluaron el programa KiVa multidimensional para toda la escuela^(6,32,33,34,38,39,40,41)^. Los 15 estudios restantes (65,2 %) evaluaron enfoques más específicos: seis intervenciones curriculares^(20,22,27,28,29,30)^, cinco intervenciones de apoyo entre pares^(24,25,26,36,37)^, tres programas virtuales o basados en computadora^(23,31,35)^ y una intervención de grupo de apoyo que involucraba a los espectadores y se enfocaba en los estudiantes víctimas^(42)^.

### Evaluación del Riesgo de Sesgo

Las puntuaciones de Downs y Black oscilaron entre 1 y 24 de un total de 28 puntos posibles (mediana: 14,0).

Ocho estudios (34,8 %) se clasificaron como de bajo riesgo de sesgo, nueve (39,1 %) como de riesgo incierto y seis (26,1 %) como de alto riesgo. El cuadro 3 presenta las puntuaciones de presentación de datos, validez externa, sesgo interno, factores de confusión y poder estadístico para cada estudio.

Axford et al.^(38)^ y Desmet et al.^(23)^ obtuvieron las puntuaciones totales más altas (24 cada uno). Los seis estudios de alto riesgo fueron los de Andreou et al.^(27)^, Karna et al.^(32)^, Salmivalli et al.^(6)^, Pack et al.^(36)^, Andreou et al.^(28)^ y Vannini et al.^(31)^.

### Efecto de las Intervenciones

Los 23 estudios contribuyeron a más de un resultado cuando correspondía: 15 evaluaron el comportamiento defensivo, 12 la perpetración de acoso escolar, 14 la victimización por acoso escolar, tres la perpetración de ciberacoso y cuatro la cibervictimización. Los recuentos narrativos a continuación se refieren a estudios únicos, mientras que el Cuadro 4 distingue los estudios únicos de las comparaciones independientes incluidas en cada metaanálisis.

**Cuadro 4:** Resumen de los nueve metaanálisis – Fortaleza, CE, Brasil, 2025.

Síntesis	Resultado y estudios incluidos	Estudios (comparaciones)	Participantes	Efecto combinado (IC del 95 %)	Valor p	I^2^
4A	Comportamiento de defensa: pedir ayuda o acudir a un adulto Merrell^(22)^; Stevens et al.^(29)^	2 (3)	827	DM -0,03 (-0,43 a 0,37)	0,88	84 %
4B	Comportamiento defensivo/positivo Desmet et al.^(23)^; Menesini et al.^(37)^; Stevens et al.^(29)^	3 (4)	1.103	DM 0,06 (-0,18 a 0,30)	0,61	68 %
4C	Prevalencia de la perpetración del acoso escolar Axford et al.^(38)^; Desmet et al.^(23)^; Nocentini y Menesini^(33)^	3 (4)	5 320	RR 0,75 (0,60 a 0,95)	0,02	0 %
4D	Prevalencia de la perpetración de ciberacoso Desmet et al.^(23)^; Williford et al.^(40)^	2 (3)	14 101	RR 0,93 (0,61 a 1,40)	0,72	38 %
4E	Cometimiento de acoso escolar, resultado continuo; grupo de Chaux de mayor duración Chaux et al.^(20)^; Karna et al.^(32)^; Karna et al.^(39)^; Nocentini y Menesini^(33)^	4 (6)	23 699	DM -0,03 (-0,04 a -0,01)	<0,0001	86 %
4F	Cometimiento de acoso escolar, resultado continuo; grupo de Chaux de menor duración Chaux et al.^(20)^; Karna et al.^(32)^; Karna et al.^(39)^; Nocentini y Menesini^(33)^	4 (6)	23 608	DM -0,03 (-0,04 a -0,01)	<0,0001	83 %
4G	Prevalencia de la victimización por acoso escolar Axford et al.^(38)^; Desmet et al.^(23)^; Salmivalli et al.^(6)^	3 (3)	9 081	RR 0,63 (0,40 a 0,99)	0,04	0 %
4H	Victimización por acoso escolar, resultado continuo; grupo de Chaux de menor duración Chaux et al.^(20)^; Karna et al.^(32)^; Karna et al.^(39)^; Nocentini y Menesini^(33)^	4 (6)	23 683	DM -0,05 (-0,08 a -0,02)	0,002	94 %
4I	Victimización por acoso escolar, resultado continuo; grupo de Chaux de mayor duración Chaux et al.^(20)^; Karna et al.^(32)^; Karna et al.^(39)^; Nocentini y Menesini^(33)^	4 (6)	23 777	DM -0,06 (-0,09 a -0,03)	0,0003	95 %

DM: diferencia media; RR: razón de riesgo; IC: intervalo de confianza; I^2^: porcentaje de variabilidad atribuible a la heterogeneidad. Los estudios se contabilizaron una sola vez en la columna de estudios. Los estratos independientes y no superpuestos a nivel de curso o centro educativo se introdujeron como comparaciones separadas. Los grupos de intervención de menor y mayor duración de Chaux et al.^(20)^ se analizaron en síntesis separadas. En las figuras 6, 7, 9 y 10, el grupo de duración pertinente de Chaux et al.^(20)^ contribuye a la estimación combinada; el grupo alternativo se muestra con una ponderación nula para documentar la estructura de los datos sin doble recuento.

En cuanto al comportamiento defensivo, siete de los 15 estudios reportaron un efecto favorable estadísticamente significativo^(24,27,29,31,32,34,42)^. Pack et al.^(36)^ describieron una tendencia favorable, pero no proporcionaron una prueba de significación estadística. Seis estudios no reportaron ningún efecto estadísticamente significativo^(22,23,25,28,35,37)^, y Karna et al.^(39)^ reportaron resultados mixtos, incluyendo una disminución en el comportamiento defensivo en un subgrupo. Los efectos variaron según el momento de la evaluación^(26,27)^, el nivel educativo^(29)^ y las características de la muestra^(31)^.

Entre los 12 estudios que evaluaron la perpetración de acoso escolar, ocho informaron reducciones estadísticamente significativas^(20,25,27,32,33,34,39,41)^, mientras que cuatro no lo hicieron^(23,28,37,38)^. Los resultados variaron según el grado o la edad^(39)^, la duración de la intervención^(20)^, el momento de la evaluación^(27)^ y si los resultados fueron reportados por los propios participantes o por sus compañeros^(32,39)^. Cinco de los ocho estudios con resultados favorables evaluaron el programa KiVa^(32,33,34,39,41)^.

Tres estudios evaluaron la perpetración de acoso cibernético^(20,23,40)^. Chaux et al.^(20)^ y Williford et al.^(40)^ reportaron reducciones significativas en al menos un análisis, con variaciones según la duración de la intervención o el grupo de grado, mientras que Desmet et al.^(23)^ no encontraron ningún efecto significativo.

Entre los 14 estudios que evaluaron la victimización por acoso escolar, ocho reportaron reducciones estadísticamente significativas^(6,30,32,33,34,39,41,42)^ y seis no^(20,23,27,28,37,38)^. En el estudio de Pfetsch et al.^(30)^, la reducción fue evidente solo cuando la calidad de la implementación fue alta. En el de van der Ploeg et al.^(42)^, se observó una mejoría a corto plazo, pero no se mantuvo, y Karna et al.^(39)^ reportaron variaciones relacionadas con la edad. Seis de los ocho estudios con resultados favorables evaluaron el programa KiVa^(6,32,33,34,39,41)^.

Cuatro estudios evaluaron la cibervictimización^(6,20,23,40)^. Salmivalli et al.^(6)^ y Williford et al.^(40)^ reportaron hallazgos favorables significativos, mientras que Chaux et al.^(20)^ y Desmet et al.^(23)^ reportaron resultados no significativos.

Un patrón descriptivo favoreció los enfoques multidimensionales: siete de las ocho evaluaciones de KiVa^(6,32,33,34,38,39,40,41)^ reportaron un efecto favorable significativo para al menos un resultado, con la excepción de Axford et al.^(38)^. Este patrón no se evaluó en un análisis formal de subgrupos y, por lo tanto, no debe interpretarse como una estimación comparativa del efecto.

### Síntesis Cuantitativa de los Estudios Incluidos: Metaanálisis

Se realizaron nueve metaanálisis de efectos aleatorios tras agrupar los estudios por constructo de resultado, tipo de datos, exposición a la intervención y momento de la evaluación: dos sobre el comportamiento defensivo (4A-4B), cuatro sobre la perpetración de acoso escolar o ciberacoso (4C-4F) y tres sobre la victimización por acoso escolar (4G-4I). El cuadro 4 presenta tanto los estudios únicos como las comparaciones independientes; los Forest plots correspondientes se muestran en las figuras 2 a 10.

**Figura 2:** Forest plot sobre el efecto de las intervenciones centradas en los testigos en el comportamiento de defensa (pedir ayuda/contactar a un adulto) – Fortaleza, CE, Brasil, 2025.

**Figura 3:** Forest plot del efecto de las intervenciones sobre el comportamiento defensivo o positivo de los testigos – Fortaleza, CE, Brasil, 2025.

**Figura 4:** Forest plot sobre el efecto de las intervenciones en la prevalencia de la perpetración de acoso escolar – Fortaleza, CE, Brasil, 2025.

**Figura 5:** Forest plot sobre el efecto de las intervenciones en la prevalencia de la perpetración de ciberacoso – Fortaleza, CE, Brasil, 2025.

**Figura 6:** Forest plot del efecto de las intervenciones sobre la perpetración de acoso escolar utilizando el grupo de intervención de mayor duración descrito por Chaux et al.^(20)^ – Fortaleza, CE, Brasil, 2025.

**Figura 7:** Forest plot del efecto de las intervenciones sobre la perpetración del acoso escolar utilizando el grupo de intervención de menor duración descrito por Chaux et al.^(20)^ – Fortaleza, CE, Brasil, 2025.

**Figura 8:** Forest plot sobre el efecto de las intervenciones en la prevalencia de la victimización por acoso escolar – Fortaleza, CE, Brasil, 2025.

**Figura 9:** Forest plot sobre el efecto de las intervenciones en la victimización por acoso escolar, utilizando el grupo de intervención de menor duración descrito por Chaux et al.^(20)^ – Fortaleza, CE, Brasil, 2025.

**Figura 10:** Forest plot del efecto de las intervenciones sobre la victimización por acoso escolar, utilizando el grupo de intervención de mayor duración descrito por Chaux et al.^(20)^ – Fortaleza, CE, Brasil, 2025.

### Metaanálisis Sobre el Comportamiento Defensivo

La síntesis 4A incluyó dos estudios y tres comparaciones independientes por nivel educativo (827 participantes). Las intervenciones enfocadas en los testigos no mejoraron significativamente la búsqueda de ayuda ni el contacto con un adulto (DM −0,03; IC del 95 %: −0,43 a 0,37; p = 0,88), y la heterogeneidad fue alta (I2 = 84 %). El Forest plot correspondiente se presenta en la Figura 2.

La síntesis 4B incluyó tres estudios y cuatro comparaciones (1 103 participantes). El efecto combinado sobre el comportamiento de defensa o el comportamiento positivo de los testigos no fue significativo (DM 0,06; IC del 95 %: −0,18 a 0,30; p = 0,61), con una heterogeneidad moderada (I2 = 68 %). El Forest plot correspondiente se presenta en la Figura 3.

Stevens et al.^(29)^ aportaron comparaciones separadas y sin superposición entre la educación primaria y la secundaria a las síntesis 4A y 4B; el estudio en sí se contó una sola vez en el recuento de estudios únicos.

### Metaanálisis Sobre la Perpetración del Acoso Escolar y el Ciberacoso

La síntesis 4C incluyó tres estudios y cuatro comparaciones (5 320 participantes). Las intervenciones enfocadas en los espectadores redujeron la prevalencia de la perpetración de acoso escolar (RR 0,75; IC del 95 %: 0,60 a 0,95; p = 0,02), sin que se observara heterogeneidad estadística (I2 = 0 %). El Forest plot correspondiente se presenta en la Figura 4.

La síntesis 4D incluyó dos estudios y tres comparaciones entre grupos de grado (14101 participantes). El efecto combinado sobre la perpetración de acoso cibernético no fue significativo (RR 0,93; IC del 95 %: 0,61 a 1,40; p = 0,72), y la heterogeneidad fue baja (I2 = 38 %). El Forest plot correspondiente se presenta en la Figura 5.

Williford et al.^(40)^ aportaron datos por separado para los alumnos de cuarto a sexto grado y de octavo a noveno grado a la síntesis 4D.

La síntesis 4E combinó cuatro estudios y seis comparaciones (23 699 participantes), utilizando el grupo de intervención de mayor duración descrito por Chaux et al.^(20)^. Las puntuaciones de perpetración de acoso fueron más bajas en los grupos de intervención (DM −0,03; IC del 95 %: −0,04 a −0,01; p < 0,0001), con una alta heterogeneidad (I2 = 86 %). El Forest plot correspondiente se presenta en la Figura 6.

La síntesis 4F incluyó los mismos cuatro estudios y seis comparaciones, pero utilizó el grupo de Chaux de menor duración (23 608 participantes). El efecto combinado fue una diferencia media (DM) de −0,03 (IC del 95 %: −0,04 a −0,01; p < 0,0001), también con alta heterogeneidad (I2 = 83 %). El Forest plot correspondiente se presenta en la Figura 7.

Karna et al.^(39)^ y Nocentini y Menesini^(33)^ aportaron estratos independientes a nivel de grado o de escuela a las síntesis 4C, 4E y 4F. Estos estratos se trataron como comparaciones separadas sin contar la publicación correspondiente como más de un estudio.

### Metaanálisis Sobre la Victimización por Acoso Escolar

La síntesis 4G incluyó a Axford et al.^(38)^, Desmet et al.^(23)^ y Salmivalli et al.^(6)^ (9,081 participantes). Las intervenciones redujeron la prevalencia de la victimización por acoso escolar (RR 0,63; IC del 95 %: 0,40 a 0,99; p = 0,04), sin que se observara heterogeneidad (I2 = 0 %). El Forest plot correspondiente se presenta en la Figura 8.

La síntesis 4H incluyó cuatro estudios y seis comparaciones (23 683 participantes) y utilizó el grupo de intervención de menor duración de Chaux et al.^(20)^. Las puntuaciones de victimización por acoso escolar fueron más bajas en los grupos de intervención (DM −0,05; IC del 95 %: −0,08 a −0,02; p = 0,002), con una alta heterogeneidad (I2 = 94 %). El Forest plot correspondiente se presenta en la Figura 9.

La síntesis 4I incluyó los mismos cuatro estudios y seis comparaciones, utilizando el grupo de Chaux de mayor duración (23 777 participantes). El efecto combinado fue una diferencia media (DM) de −0,06 (IC del 95 %: −0,09 a −0,03; p = 0,0003), con alta heterogeneidad (I2 = 95 %). El Forest plot correspondiente se presenta en la Figura 10.

Karna et al.^(39)^ y Nocentini y Menesini^(33)^ aportaron estratos independientes por nivel educativo a las síntesis 4H y 4I. El grupo de intervención de duración alternativa de Chaux se excluyó de cada estimación combinada respectiva para evitar el doble recuento.

En todas las síntesis, la evidencia respaldó reducciones en la perpetración y la victimización por acoso escolar tradicional. Los efectos combinados no fueron significativos para la perpetración de acoso cibernético ni para el comportamiento defensivo. Las síntesis de resultados continuos mostraron una heterogeneidad de moderada a alta (I2 = 68 %–95 %), y el reducido número de estudios impidió una exploración formal confiable de sus causas.

### Clasificación de la Certeza de la Evidencia

La certeza según el sistema GRADE fue alta para la prevalencia de la perpetración de acoso escolar (RR 0,75) y la prevalencia de la victimización por acoso escolar (RR 0,63). La certeza fue muy baja para las siete síntesis restantes debido a una combinación de limitaciones de los estudios, inconsistencia e imprecisión.

El cuadro 5 presenta la evaluación completa de la certeza para cada metaanálisis. No se evaluó formalmente el sesgo de notificación, ya que cada síntesis contenía menos de 10 estudios únicos; no se rebajó ninguna calificación de certeza únicamente sobre la base de un gráfico “funnel” no interpretable.

**Cuadro 5:** Evaluación GRADE de la certeza de los nueve metaanálisis – Fortaleza, CE, Brasil, 2025.

Resultado (síntesis)	Estudios (comparaciones)	Participantes	Efecto (IC del 95 %)	Motivos de la rebaja de la certeza/valoración	Certeza
4A Defensa: pedir ayuda/ponerse en contacto con un adulto	2 (3)	827	DM -0,03 (-0,43 a 0,37)	Limitaciones del estudio; inconsistencia muy grave (I^2^ = 84 %); imprecisión grave	Muy baja
4B Comportamiento defensivo/positivo	3 (4)	1 103	DM 0,06 (-0,18 a 0,30)	Limitaciones del estudio; inconsistencia grave (I^2^ = 68 %); imprecisión grave	Muy baja
4C Prevalencia de la perpetración de acoso escolar	3 (4)	5 320	RR 0,75 (0,60 a 0,95)	No se han identificado problemas graves en los ámbitos evaluados	Alta
4D Prevalencia de la perpetración de ciberacoso	2 (3)	14 101	RR 0,93 (0,61 a 1,40)	Limitaciones del estudio; inconsistencia; imprecisión grave	Muy baja
4E Acoso escolar, grupo de mayor duración	4 (6)	23 699	DM -0,03 (-0,04 a -0,01)	Limitaciones del estudio; inconsistencia muy grave (I^2^ = 86 %)	Muy baja
4F Acoso escolar, grupo de menor duración	4 (6)	23 608	DM -0,03 (-0,04 a -0,01)	Limitaciones del estudio; inconsistencia muy grave (I^2^ = 83 %)	Muy baja
4G Prevalencia de la victimización por acoso escolar	3 (3)	9 081	RR 0,63 (0,40 a 0,99)	No se han identificado problemas graves en los ámbitos evaluados	Alta
4H: Victimización por acoso, grupo de menor duración	4 (6)	23 683	DM -0,05 (-0,08 a -0,02)	Limitaciones del estudio; inconsistencia muy grave (I^2^ = 94 %)	Muy baja
4I Victimización por acoso escolar, grupo de mayor duración	4 (6)	23 777	DM -0,06 (-0,09 a -0,03)	Limitaciones del estudio; inconsistencia muy grave (I^2^ = 95 %)	Muy baja

IC: intervalo de confianza; DM: diferencia media; RR: razón de riesgo; I^2^: heterogeneidad. Se contabilizaron como comparaciones los estratos independientes y no superpuestos. No se realizaron pruebas formales del efecto de los estudios pequeños, ya que cada síntesis contenía menos de 10 estudios únicos.

## DISCUSIÓN

Esta revisión encontró que las intervenciones escolares que incorporaban estrategias para los testigos se asociaron con reducciones en la perpetración y la victimización del acoso escolar tradicional. Por el contrario, las estimaciones combinadas no mostraron efectos significativos sobre la perpetración del acoso cibernético ni sobre el comportamiento defensivo. Dado que varias síntesis de resultados continuos mostraron una alta heterogeneidad, esas estimaciones deben interpretarse con cautela.

Siete de las ocho evaluaciones de KiVa reportaron un efecto favorable para al menos un resultado, lo que sugiere que los programas multidimensionales que abarcan toda la escuela pueden ofrecer un contexto útil para los componentes dirigidos a los testigos^(6,32,33,34,39,40,41)^. Sin embargo, se trató de un patrón descriptivo más que de una comparación formal entre programas. La revisión no realizó un análisis de subgrupos, y las diferencias en los participantes, la implementación, la medición de resultados y el seguimiento podrían explicar el patrón observado.

Los dos metaanálisis de prevalencia indicaron reducciones en la perpetración y la victimización por acoso escolar tradicional autoinformadas y se calificaron como evidencia de alta certeza. La certeza de todas las demás síntesis fue muy baja. Por lo tanto, las estimaciones significativas de resultados continuos para la perpetración y la victimización no deben interpretarse como igualmente sólidas, especialmente porque el I2 osciló entre el 83 % y el 95 % en esos análisis.

Un metaanálisis anterior de 12 estudios y 12 784 participantes reportó un aumento moderado en el comportamiento defensivo de los espectadores (g de Hedges = 0,20, IC del 95 %: 0,11 a 0,29; p < 0,001), con efectos más marcados entre los estudiantes de mayor edad^(12)^. Las diferencias con respecto a los presentes hallazgos pueden deberse a que el período de revisión fue anterior, a que se incluyeron intervenciones más amplias y a que se utilizaron definiciones y medidas diferentes del comportamiento de defensa.

La defensa es un comportamiento complejo influenciado por factores individuales, de los compañeros, familiares, del salón de clases y de la escuela^(43,44)^. Una intervención puede reducir el refuerzo del acoso escolar o aumentar la búsqueda de ayuda sin producir un aumento medible en la defensa directa. Esto podría explicar por qué se observaron reducciones en la perpetración y la victimización incluso cuando los resultados combinados de la defensa no fueron significativos.

El patrón diferencial entre los resultados sugiere que los componentes relacionados con los espectadores podrían actuar menos a través de aumentos en la defensa directa y más a través de cambios en las normas entre pares, un menor refuerzo social de la agresión y un mayor uso de la búsqueda segura de ayuda y el apoyo de los adultos. Esta interpretación concuerda con el patrón descriptivo favorable observado en programas multidimensionales, aunque no se evaluó la eficacia comparativa. En consecuencia, la implementación debería combinar la educación de los estudiantes con la capacitación del personal, los protocolos de respuesta institucional y el monitoreo del clima escolar. Las enfermeras de programas escolares y otros profesionales de la salud pueden contribuir identificando las consecuencias psicológicas y somáticas relacionadas con el acoso escolar, apoyando a los estudiantes afectados y a sus familias, y coordinando las vías de derivación con los equipos educativos.

La ausencia de un efecto combinado significativo en el ciberacoso puede reflejar el reducido número de estudios elegibles y la atención limitada a las características específicas de los contextos en línea, incluyendo el anonimato, la rápida difusión y la persistencia del contenido digital. El amplio intervalo de confianza para la perpetración del ciberacoso era compatible tanto con la presencia de beneficios como con la ausencia de ellos, por lo que el resultado debe considerarse inconclusivo en lugar de una evidencia de ineficacia.

En cuanto a la práctica y las políticas, la educación de los espectadores puede incorporarse a los programas escolares integrales contra el acoso escolar, pero su implementación debe incluir capacitación del personal, monitoreo de la fidelidad al programa, vías claras de derivación y evaluación de resultados. El contenido del ciberacoso y las formas seguras de buscar ayuda deben abordarse de manera explícita, en lugar de dar por sentado que simplemente se transfieren de los programas tradicionales contra el acoso escolar.

Las fortalezas de esta revisión incluyen una búsqueda amplia en bases de datos y en literatura gris, la ausencia de restricciones de idioma o fecha, procedimientos de revisión independientes y una evaluación explícita de la certeza. Persisten limitaciones importantes. Seis estudios se clasificaron con alto riesgo de sesgo y nueve con riesgo incierto; estas limitaciones pueden exagerar o atenuar los efectos y contribuyeron a rebajar la calificación de varios resultados. Los formatos de intervención y las medidas de resultado variaron sustancialmente, y el reducido número de estudios en cada síntesis impidió realizar análisis formales de subgrupos, metarregresión, sensibilidad o sesgos de publicación confiables. Además, múltiples informes aportaron estratos independientes por nivel escolar, por lo que los totales de participantes en los metaanálisis no deben sumarse como un recuento de individuos únicos. La mayoría de los estudios se realizaron en entornos con un alto Índice de Desarrollo Humano, lo que puede limitar la transferibilidad a otros contextos.

## CONCLUSIÓN

Las intervenciones escolares que incluyen estrategias enfocadas en los espectadores pueden reducir la perpetración y la victimización tradicionales del acoso escolar, con evidencia de alta certeza para los dos resultados de prevalencia. La evidencia sobre el comportamiento defensivo y el ciberacoso sigue siendo muy incierta, y las estimaciones de resultados continuos se vieron afectadas por una heterogeneidad sustancial.

Las escuelas y los responsables de las políticas de salud y educación deberían integrar componentes dirigidos a los espectadores en programas integrales, al tiempo que monitorean la implementación y la seguridad. Los estudios futuros deberían utilizar diseños controlados con el poder estadístico adecuado, definiciones estandarizadas de los resultados, análisis preespecificados, un seguimiento más prolongado y muestras de entornos socioeconómicos y culturales diversos, con una evaluación explícita del acoso cibernético y de las diferentes formas de defensa.

## Data Availability

Los datos extraídos para esta revisión y los Forest plots que respaldan los metaanálisis se proporcionan en este artículo.
